# Ultrathin seed wing with heterogeneous structures for highly efficient dispersal of African tulip tree

**DOI:** 10.1093/nsr/nwag132

**Published:** 2026-03-03

**Authors:** Jiangkun Wei, Mingshen Wu, Yongtao Dai, Peiyu Cao, Hao Yang, Zheng Chen, Jinzhao Yang, Zhigang Wu, Stanislav Gorb, Jianing Wu

**Affiliations:** School of Aeronautics and Astronautics, Sun Yat-sen University, Shenzhen 518107, China; School of Advanced Manufacturing, Sun Yat-sen University, Shenzhen 518107, China; School of Aeronautics and Astronautics, Sun Yat-sen University, Shenzhen 518107, China; School of Aeronautics and Astronautics, Sun Yat-sen University, Shenzhen 518107, China; School of Aeronautics and Astronautics, Sun Yat-sen University, Shenzhen 518107, China; School of Aeronautics and Astronautics, Sun Yat-sen University, Shenzhen 518107, China; School of Aeronautics and Astronautics, Sun Yat-sen University, Shenzhen 518107, China; School of Aeronautics and Astronautics, Sun Yat-sen University, Shenzhen 518107, China; Functional Morphology and Biomechanics, Zoology Department, Kiel University, Kiel 24118, Germany; School of Aeronautics and Astronautics, Sun Yat-sen University, Shenzhen 518107, China; School of Advanced Manufacturing, Sun Yat-sen University, Shenzhen 518107, China

**Keywords:** African tulip tree, winged seed, heterogeneous structure, wind dispersal, passive micro-flier

## Abstract

To colonize new habitats, plant seeds have evolved a variety of specialized structures for wind dispersal. The African tulip tree, identified as one of the world’s 100 worst invasive alien species, features filmy winged seeds predominantly dispersed by wind. Herein, we found that most of the wing region is single-cell-layer thick with the thinnest part measuring only 0.4 μm, enabling the wing to cover 90% of the seed’s size while constituting only 25% of the total mass. We revealed that such an ultrathin wing is reinforced by a series of heterogeneous vein-like structures that maintain the wing extension even in turbulent airflow and can resist circumferential fracture to minimize detrimental impacts on aerodynamic performance. The filmy wing is also able to conformably attach to uneven wet substrates, enhancing the chance of locating on water-rich soils for seed germination. The unique geometry and material composition in the seed wing present an elegant natural solution in balancing light weight, proper stiffness, and directional toughness suited for varied environments. Inspired by the natural seed, we designed a passive micro-flier with a customizable wing that can realize multiple functions, illuminating new possibilities in large-scale aerial delivery for wide-range sampling and environmental monitoring.

## Teaser

Uncovering how the ultrathin wing structure of the African tulip tree seed balances lightness, stiffness, and toughness inspires a new design of robust and versatile micro-fliers for large-scale environmental monitoring.

## INTRODUCTION

Seed dispersal through the air typically relies on extremely lightweight materials to remain airborne, yet can still achieve multifunction through specialized designs that enable adaptation to alternating environments [1]. Generally, there are two kinds of seeds that can fly in the wind: (i) pappose seeds that feature filamentous wings and exhibit a parachuting flight pattern, and (ii) winged seeds, which are structured with an appendant wing that enables gliding, straying, and spinning [2]. Compared to most filamentous diaspores, the winged seeds are usually capable of loading much heavier mass [1] and are striking in long-distance dispersal capability [3]. In nature, winged seeds are found in many unrelated taxa [4], suggesting that the use of wings represents a convergent solution for anemochory dispersal [5], which could provide an excellent example of transporting matter in the air using passive strategies. Studies of winged seeds have strengthened our understanding of plant ecology, the underlying mechanism of fluid-structure interaction [6], and have allowed for the recent emergence of passive micro-fliers served for aerial delivery [7–9].

**Figure 1.**
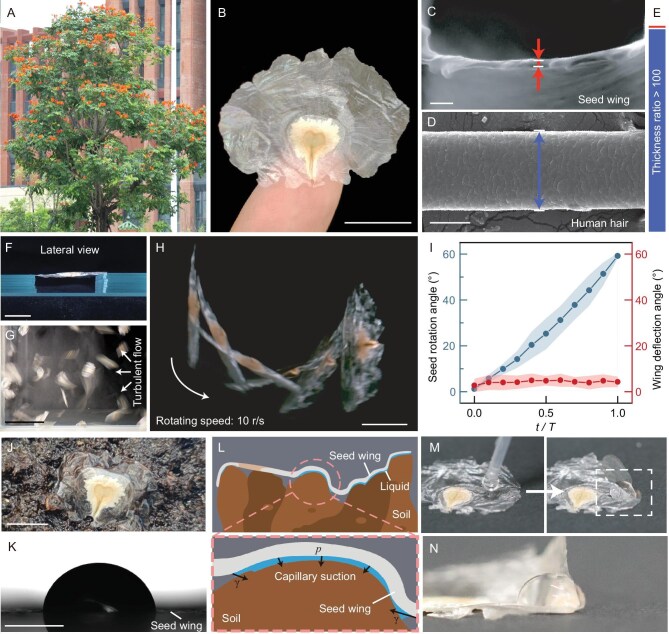
Performance of the ultrathin seed wing of the African tulip tree. (A) African tulip tree with its striking red flowers. (B) Seed attached on a fingertip, featuring a heart-shaped core in the middle and an extending filmy wing. Scale bar, 10 mm. (C) Scanning electron microscopy (SEM) image showing the cross-sectional view of the seed wing. Scale bar, 2 μm. (D) Lateral view of a human hair for size comparison. Scale bar, 50 μm. (E) Color bar illustrating the thickness ratio between the human hair (blue) and the wing film (red). (F) Lateral view showing that only two edges of the wing can fully support the suspended seed. Scale bars, 10 mm. (G) Experimental setup for observing the seed wing in turbulent airflow. (H) Superposed frames from the high-speed video showing a typical seed rotating in the turbulence with its wing barely deformed. Scale bar, 10 mm. (I) Time-variation graphs of the seed rotation angle and the wing deflection angle, respectively, when the seed is fast moving in the turbulence (*n* = 10). (J) Seed on wet soil with its wing significantly deformed. Scale bar, 10 mm. (K) The flattened seed wing placed with a water droplet exhibiting hydrophilicity. (L) Schematics showing the wing constrained by the capillary suction on the wet soil. (M) Seed wing can spontaneously curve upon contacting with a water droplet. (N) Magnified lateral view showing the largely deformed wing when contacting the water droplet.

The evergreen tree *Spathodea campanulata* (Fig. 1A), commonly called the African tulip tree, possesses filmy winged seeds that are mainly dispersed by wind [10]. This species is renowned for its rapid growth and is listed among the 100 world’s worst invasive alien species, with only 30 of them being terrestrial plants [11]. Native to the west coast of Africa [12], the African tulip tree has now spread globally, even to the Caribbean and Pacific Islands [13], and we anticipate its seeds to have remarkable adaptability for long-distance dispersal. In fact, the seeds of the African tulip tree released from 20 m height are able to fly >600 m away through a breeze [10], surpassing the capability of any current man-made passive micro-flier [9]. Furthermore, the seed of the African tulip tree can thrive in a variety of environments, including roadsides, deforested lands [10], and abandoned agricultural lands in tropical regions [14,15] and can colonize irregular slopes steeper than $45^\circ $ with both poor and excessive soil drainage [10,16], which strongly indicates its prominent adaptability to various environments.

Generally, the capability to sustain flight for extended periods is essential for effective long-distance dispersal [17]. In this study, we found that the filmy wing of the African tulip tree seed is extremely thin (∼0.4 μm) compared to its wing span (>20 000 μm), which reduces its overall weight and facilitates its ability to stay in the air, leading to an average falling velocity of ∼0.3 m/s. As comparison, the natural maple samara, a typical winged seed that can autorotate in the air for extended flight, descends at a speed of 0.72 m/s [18]. We note that the ultrathin wing of the seed may be prone to deformation, folding, or even fracturing in turbulent airflow due to its extreme construction, which could severely impact its aerodynamic performance. Although the aerodynamics of the general winged seed have been extensively examined [2,6,19,20], the role of wing structure and material composition in flight stability remains poorly explored. Beyond flight, germination in the soil is also an indispensable determinant of invasion success [10]. A seed must remain in a particular microsite long enough for imbibition, respiration, and synthesis of nucleic acids and proteins to germinate [21,22], which generally requires a longer duration than the time in air dispersal. In natural environments, seeds could be moved by wind, rainwater, or predators, which can impede the absorption of water and nutrition [22]. While substantial efforts have been directed toward the flight of the winged seed, less attention has been given to their subsequent fate after landing, particularly the mechanism of anchorage to the soil [3].

In this study, we aim to investigate how the filmy wing of the African tulip tree supports flight and adapts to substrates upon landing. Our findings reveal that the ultrathin wing of the seed has a specialized vein-like reinforcement composed of heterogeneous materials, the design of which provides a natural solution balancing light weight, proper deformability, and fracture resistance, enhancing both environmental adaptability and multifunctionality. Inspired by the natural seed wing, we further design and fabricate a bioinspired micro-flier composed of heterogeneous materials, which can achieve multifunctionality while remaining lightweight.

## RESULTS

### Multifunction of the seed wing

The seed was flat, with a heart-shaped core in the middle and a filmy wing that radiated ∼1 cm from the round edge of the core, but only extended 1–3 mm from the sharp side (Fig. 1B). The seed was approximately elliptical in shape, with a span of 2.8 ± 0.5 cm along the long axis, a height of 1.6 ± 0.2 cm along the short axis, and a weight of 5.0 ± 1.5 mg (sample number, *n* = 15). The core itself measures 0.7 ± 0.1 cm in width, 0.8 ± 0.1 cm in length, and 306 ± 14 μm in thickness, with a mass of 3.8 ± 1.2 mg and a surface area of 0.4 ± 0.1 cm^2^. The surface area of the seed wing was 3.6 ± 0.9 cm^2^, covering 90% of the total seed surface area. A comparatively large seed wing area should provide sufficient aerodynamic force during flight since a positive correlation between the seed surface area and dispersal distance could be observed (Fig. S1). Notably, we found that the wing film is extremely thin with a minimum thickness of 414 ± 13 nm (*n* = 20) (Fig. S2), about 100 times thinner than a human hair (Fig. 1C, D, and E). Such an ultrathin structure results in the wing mass being only 1.25 ± 0.34 mg (*n* = 15), taking ∼25% of the total seed mass.

When the seed was only supported at two edges, the filmy wing was still able to maintain its extended shape, indicating sufficient stiffness to support its self-weight (Fig. 1F). We deposited a batch of seeds (*n* = 100) in a transparent box and subjected them to turbulent airflow, where the seeds exhibited highly random movements including rapid translation and rotation (Fig. 1G, Video S1). Despite reaching a rotational speed of ∼10 revolutions per second (r/s), the wing experienced only minimal deformation, with a deflection angle of <$5^\circ $ (*n* = 10; Fig. 1H, I), demonstrating excellent stability as an aerodynamic structure.

However, the thin wing would deform significantly as the seed landed on wet soil (Fig. 1J). This deformation was likely due to the wing’s hydrophilic surface, which has a water contact angle of ${75}^\circ \pm {4}^\circ $ (*n* = 8, Fig. 1K). The hydrophilic surface allowed a liquid bridge to form between the seed wing and the wet soil, which pulled the thin wing towards the soil by capillary suction (Fig. 1L). Remarkably, the wing could spontaneously curve >$90^\circ $ upon placing a water droplet (Fig. 1M, N, Video S2). The deformed hydrophilic surface enabled the seeds to remain in contact with the wet soil in windy conditions, greatly promoting the attachment rate compared to seeds on the dry substrate (Fig. S3). Even after germination, the wing continued to anchor to the soil and further contributed to stabilizing the plumule (*n* = 10) (Fig. S3), exhibiting the multifunctional utility of the wing.

### Wing microstructure and material compositions

**Figure 2.**
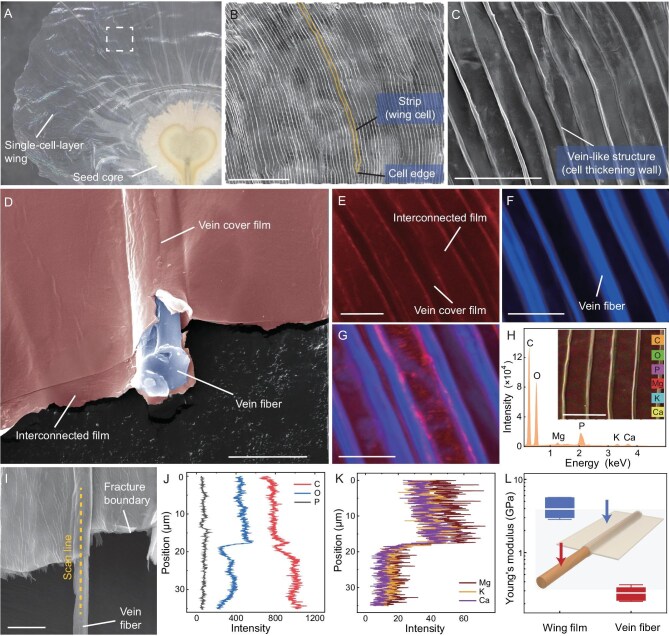
Heterogeneous structures of the seed wing. (A) Magnified view of the seed surface. (B) SEM image of the rectangular region in (A) showing dense slender strips on the seed wing, identified as elongated wing cells called tracheoids. Scale bar, 500 μm. (C) Magnified view showing the wing cells evenly spaced on the surface of the filmy wing. Scale bar, 100 μm. (D) Fractured wing exposing the vein fiber inside the vein-like structure. Scale bar, 10 μm. (E) Confocal laser scanning microscopy (CLSM) image of Basic-Fuchsin-stained seed wing, indicating the presence of lignin in flattened film and vein cover film. (F) CLSM image of a Calcofluor-White-stained seed wing, indicating the presence of cellulose or other beta-1,4-linked carbohydrates in the vein fiber. (G) Superposed image of Basic Fuchsin and Calcofluor White sequentially stained seed wing, indicating distinct material compositions between wing film and vein fiber. Scale bars in (E)–(G), 25 μm. (H) Energy dispersive X-ray spectroscopy (EDS) element spectrum of a wing section. Inset is the superposed image of different elements in the scanning section. Scale bar, 50 μm. (I) EDS scan line section on the fractured wing surface crossing from the vein cover film to the exposed inner fiber. Scale bar, 10 μm. (J) Distributions of oxygen, carbon, and phosphorus along the EDS scan line. (K) Distributions of magnesium, potassium, and calcium along the EDS scan line. (L) Young’s moduli of the wing film and the vein fiber measured by AFM. The whiskers denote the maximum and minimum tested values.

To the best of our knowledge, the detailed morphology, material composition, and functions of the seed wing of the African tulip tree remain unexplored. The wing first extends from the edge of the seed core, forming fringes with several-cell-layers-thick (typically two or three cell layers), and further extends outward to create a single-cell-layer-thick wing that occupies ∼81% of the wing area (*n* = 6, Fig. 2A). The cell arrays constructing the wing elongate in a radial direction, which have been identified as ‘tracheoid’ in the Bignoniaceae family [23]. The tracheoid cells can reach up to 1.9 ± 0.5 mm in length, but only have an average width of 26.6 ± 3.5 μm (*n* = 10) (Fig. 2B, C). Unlike tracheids used for transporting liquid in the xylem, these tracheoid cells lack perforation plates of vessel elements and hence should have different functions. The magnified view shows that the flattened film on the wing consists of two layers, which should be the only two layers of the cell wall (Fig. 2D). The lateral wall shared between two tracheoid cells thickens to 5.3 ± 1.3 μm, forming a vein-like structure (Fig. 2D). We discovered that the interior of the vein is filled by a continuous fiber, with a diameter of 4.4 ± 0.4 μm (*n* = 10).

The functions of the plant structures are not only determined by their complex architecture, but also by their specialized material compositions [24]. The cell walls in plants are made up of just four basic building blocks: cellulose, hemicellulose, lignin and pectin, and these compositions can be visualized by specialized fluorescent stains [25,26]. Under confocal laser scanning microscopy (CLSM), we found that only the flattened film and the vein cover film could be stained by Basic Fuchsin, indicating that the film structure is primarily composed of lignin (Fig. 2E) [25]. In contrast, only the vein fibers exhibited blue fluorescence when the wing was stained with Calcofluor, suggesting the fibers are composed of cellulose or other beta-1,4-linked carbohydrates (Fig. 2F) [25]. When Basic Fuchsin and Calcofluor were used sequentially to stain the seed wing, the overlaid fluorescence CLSM images showed the interconnected film region predominantly red, while the fiber region was mainly blue, which strongly suggested that the seed wing is composed of heterogeneous materials (Fig. 2G). Additionally, both the film and fiber regions could be stained with Nile Red, indicating the existence of suberin, which would covalently bind to other macromolecules and form a protective barrier in the plant cell wall (Fig. S4A).

Under energy dispersive X-ray spectroscopy (EDS), we not only found the distinct distribution of oxygen (39.3%, in atomic%), carbon (59.1%) and phosphorus (0.4%) on the vein-like structures and the connected films (*n* = 3, Fig. 2H, Fig. S4B–D), confirming different organic compounds, but also discovered trace amounts of metallic elements that evenly distributed on the wing, including magnesium (0.35%), potassium (0.37%), and calcium (0.47%) (*n* = 3, Fig. 2H, Fig. S4E–G). The EDS scan line across the vein cover film and the exposed inner fiber revealed that those metallic elements existed mostly on the wing film (Fig. 2I–K), which may serve for cell growth and can potentially increase film rigidity [27,28]. With atomic force microscopy (AFM), we indeed found that the different material components of the wing film and the vein fiber led to their distinct mechanical properties. The average Young’s modulus of the film is ${E}_f = 3925{\mathrm{\ MPa}}$ while the vein inner fiber has an average Young’s modulus of ${E}_{in} = 280{\mathrm{\ MPa}}$, being 14 times softer than the film (*n* = 7, Fig. 2L). After the wing had been immersed in a fluorescein solution for over 12 h, no liquid could be found entering the vein through the capillary effect or being absorbed in the film via the hygroscopic effect (Fig. S4H). The seed wing could maintain its extended shape even after floating on the water for 10 d until germination (Fig. S4I), which precluded the possibility of the vein transporting water or other nutrition and suggested its purely mechanical use.

### Deformability of the seed wing

**Figure 3.**
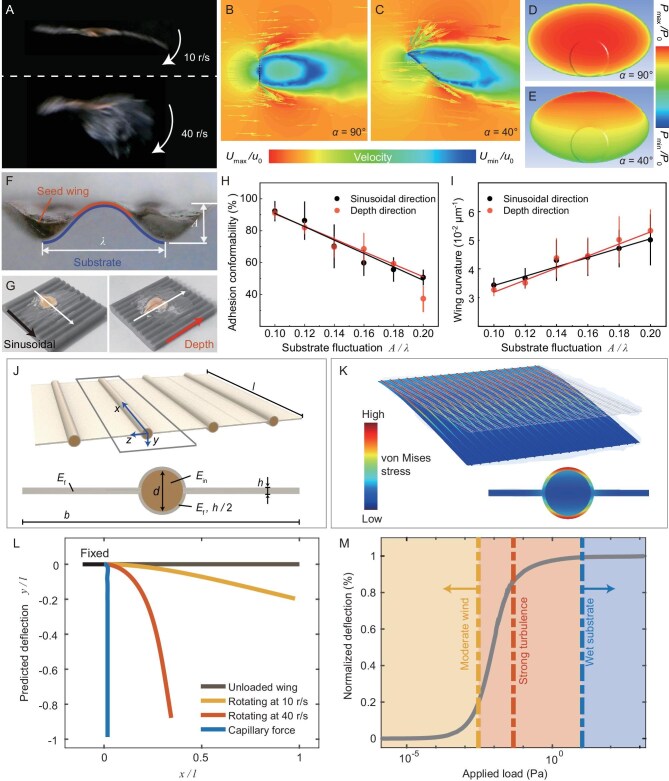
Deformability of the seed wing structure. (A) High-speed frames of the seed rotating in turbulence at a speed of ∼10 r/s (upper image) and ∼40 r/s (lower image). (B) and (C) demonstrate the velocity field of the airflow around the seed model from computational fluid dynamics (CFD) simulations at attack angles of $\alpha = 90^\circ $ and $\alpha = 40^\circ $, respectively. (D) and (E) show the pressure contour on the surface of the CFD seed model at attack angles of $\alpha = 90^\circ $ and at $\alpha = 40^\circ $, respectively. (F) Lateral view of the seed placed on a pre-wetted sinusoidal curvature substrate after overnight desiccation. Red and blue curves mark the contact length of the seed wing *L*_wing_ and the substrate ${L}_{sub}$, respectively. (G) Seed sample placed along the sinusoidal direction (black) or the depth direction (red) of the substrate, respectively. (H) Adhesion conformability, represented as the ratio *L*_wing_/*L*_sub_ plotted against the substrate fluctuation. (I) Average curvature of the deformed wing film in one sinusoidal wavelength, showing variations against the substrate fluctuation. (J) Schematic representing a geometrically simplified wing section containing four strip units. Inset displays the key mechanical parameters of the cross-section of one strip unit. (K) Deformation contour from the finite element analysis of a wing model with 20-strips under uniform pressure load. Inset shows a magnified view at the fixed end of a single wing strip, with the color bar indicating the magnitudes of von Mises stresses. (L) Theoretical model predictions of wing profiles in scenarios of rotating at 10 r/s and 40 r/s, and adhering to the wet soil, respectively. (M) Correlation between maximum wing deflection and the applied load as predicted by the ANCF model. The yellow, red, and blue dashed lines represent the applied loads on the wing in the cases of rotating at 10 r/s and 40 r/s, and adhering to the wet soil, respectively. The light yellow, light red, and light blue background represent the range of the external loads that the seed may experience under mild to moderate wind, strong turbulence, or on the wet substrate, respectively.

The heterogeneous stiffness of the wing components enables the seed to remain extended during the natural scenario of rotating flight in the air. However, high-speed video indicated that if the external force was extremely large, such as the case when the seed rotates at 40 r/s, the seed wing could undergo significant deformation (Fig. 3A, Video S3). Computational fluid dynamics (CFD) simulations were performed to estimate the aerodynamic pressure endured by the seed wing during flight (Fig. S5). The results demonstrated that at different angles of attack, the incoming airflow around the seed edge rapidly changed in both speed and direction (Fig. 3B, C). The drag on the seed increases monotonically as the attack angle rises, while the lift initially enhances with respect to the attack angles from $0^\circ $ to $40^\circ $, and then keeps dropping down at further inclined angles (Fig. S5). The alteration in airflow around the leading edge of the seed is more abrupt than that at the trailing edge, resulting in asymmetrically distributed pressures on the seed surface, which generate a torque on the overall structure and ultimately causes the seed to rapidly rotate (Fig. 3D, E).

Another case in natural scenarios is when the seed wing endures capillary force after landing on wet soil (Fig. S6). Upon contacting the wet surface of our designed sinusoidal platform, the seed film would spontaneously curve to conformably attach to the wet surface, and the wing would not recover its original flattened shape after desiccation on the platform overnight (Fig. 3F, G). We found that the adhesion conformability of the filmy wing, defined as the ratio ${L}_{wing}/{L}_{sub}$, would decrease as the fluctuation of the substrate increased, while the average curvature of the deformed wing would increase to adapt to the fluctuated substrate (Fig. 3H, I). Whether the seed’s symmetry axis was aligned with the depth direction or with the sinusoidal direction of the substrates, there was no significant difference in curvature change, suggesting that the veins had a negligible effect on retaining the wing shape when enduring the surface tension of water (Fig. 3G, I).

To analyze the deformability of the wing structure, we regarded a wing section as a series of geometrically simplified strips connected along their sides (Fig. 3J, Fig. S7). Each strip consisted of two lateral films with thickness *h*, width *b*, and length *l*, representing the interconnected film of the wing. A cylindrical rod with diameter *d* is located at the center of the film plane and is wrapped by $h/2$ thick film, forming the vein structure with a total diameter $D = d + h$. When applying uniform pressure on a 20 strip-connected wing section, our finite element analysis showed that most of the stresses are borne by the cover film of the vein during bending, indicating the stiffening use of the vein structure (Fig. 3K, Fig. S7). The inner fiber, with its significantly reduced elastic modulus, does not directly contribute to the wing’s stability. However, it may help to support the peripheral film of the cylindrical vein, which should bear the majority of the bending stress during deformation (Fig. 3K).

To describe the deformability of the wing under various loading conditions, especially for the large deformation situation, we established an analytical model based on absolute nodal coordinate formulation (ANCF, Methods, Figs S8–S10). The bending stiffness of a single strip unit can be expressed as (Fig. 3J):


\begin{eqnarray*}
B &=& \left[ {\frac{{\left( {b - h - d} \right){h}^3}}{{12}} + \frac{\pi }{{64}}{\mathrm{\ }}\left( {{D}^4 - {d}^4} \right)} \right]\\
&&\times{E}_f + \frac{\pi }{{64}}{d}^4{E}_{in}.
\end{eqnarray*}

The calculation for the model adopted the physical parameters of the real seed wing and its validity was confirmed by comparing it with the finite element analysis. The aerodynamic loads were estimated according to the CFD results and the capillary suction was estimated via the Young–Laplace equation (Figs S5, S6). Our ANCF model showed that the seed wing with the current structural design and mechanical property distribution enabled it to slightly curve while rotating at a speed of 10 r/s, significantly bend when the rotating speed reached 40 r/s, and completely deform by applying capillary force (Fig. 3L), which well aligned with the experimental observations. The correlation between the wing’s maximum deflection and the wide spectrum of the applied loads was further established to illustrate that the current design of the seed wing is capable of remaining extended—at least partially—to withstand aerodynamic forces, even in turbulent airflow (Fig. 3M). However, if the external force is comparatively large, e.g. capillary forces, the wing fails to retain its shape (Fig. 3M).

### Fracture resistance of the seed wing

**Figure 4.**
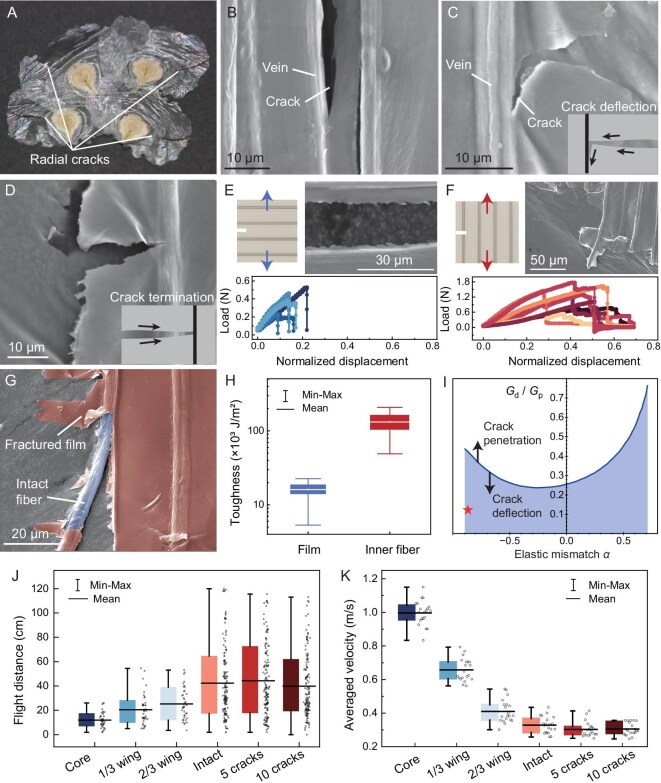
Directional fracture resistance of the seed wing. (A) Seed wings suffering from turbulent airflow showing fracture patterns with cracks only radially distributed. (B) Damaged wing with cracks propagating along the veins. (C) Damaged wing with a crack deflected by the vein. (D) Damaged wing with a crack terminated by the vein-like structure. (E and F) Tensile force with respect to loading displacement when the wing sample is stretched perpendicular (blue) and parallel (red) to the strips, respectively. Inset SEM images show the fracture patterns in each stretching direction. (G) Damaged wing with fractured vein cover film but intact inner fiber. (H) Toughness of the wing film (blue) and inner fiber (red) characterized by energy release rate (J/m^2^). Whiskers denote the maximum and minimum values. (I) Theoretical curve showing the critical ratio of energy release rates for crack deflection versus penetration, as a function of elastic mismatch $\alpha $ of the wing structures. The red star denotes the toughness ratio ${\Gamma }_f/{\Gamma }_{in}$ of the film versus the inner fiber. (J) Seed dispersal distance in windless conditions with respect to different fracture patterns of the seed wing. (K) Average falling velocity in windless conditions with respect to different fracture patterns of the seed wing.

Beyond stiffness, toughness—defined as a material’s ability to resist fracture—is another crucial mechanical property. In most artificial materials, stiffness and toughness are mutually exclusive [29,30]; stress tends to concentrate in stiffer regions, often leading to brittle fracture. This poses a significant challenge for seeds, as the natural constituents available to compose their wings are inherently limited [31]. The extremely thin wing was seemingly brittle and cracks could be easily spotted on the seeds that had just suffered intense flight (Fig. 4A, Video S4). Notably, however, only radially distributed cracks were observed on the wing (Fig. S11A). SEM images revealed that natural cracks propagated exclusively on the wing film along the vein’s axial direction (Fig. 4B), while circumferential cracks were deflected by the vein to align with this same longitudinal path (Fig. 4C), thus exhibiting directional toughness. Crack propagation could also be effectively arrested either upon perpendicular impingement on a vein or at vein intersections (Fig. 4D, Fig. S11B).

We characterized the toughness of the wing film ${\Gamma }_f$ and inner fiber ${\Gamma }_{in}$ by energy release rate, which measures the energy consumption per unit of fracture surface area (see Methods). When tension was applied perpendicular to the vein axis, cracks consistently propagated along the vein direction, resulting in smooth separation (Fig. 4E). Conversely, under parallel tension, crack propagation required significant elongation and followed a tortuous path across the veins (Fig. 4F), indicating higher energy dissipation. In some parallel tensile tests, cracks traversing the vein structure left the inner fiber intact (Fig. 4G), demonstrating the fiber’s superior fracture resistance. Experimental results confirmed that the toughness of the brittle film is significantly lower than that of the inner fiber, yielding a toughness ratio of ${\Gamma }_f/{\Gamma }_{in} = 0.12$ (Fig. 4H).

To explain the patterns of radially distributed cracks and the preservation of veins in natural wings, we analyzed the crack propagation behavior at the film–vein interface. We modeled a wing section as a thin plate where a crack approaches the interface between the film and the vein fiber at a right angle (Fig. S11C, D). The path of the crack is determined by comparing the energy release rate for deflection along the interface ${G}_d$ versus penetration into the fiber ${G}_p$. The criterion for crack deflection is governed by the ratio of the energy release rates relative to the ratio of material toughness [32,33]


\begin{eqnarray*}
\frac{{{\Gamma }_f}}{{{\Gamma }_{in}}} < \frac{{{G}_d}}{{{G}_p}},
\end{eqnarray*}

where the critical ratio ${G}_d/{G}_p$ is a function of elastic mismatch between the film and the vein fiber (Fig. 4I), which, assuming both are isotropic elastic materials, can be characterized by Dundurs’ parameter $\alpha = ( {{E}_{in} - {E}_f} )/( {{E}_{in} + {E}_f} )$ in the plane stress problem [34]. At the calculated elastic mismatch for the wing, $\alpha \cong - 0.87$, the toughness ratio falls well below the theoretical critical threshold for crack penetration of the fiber (Fig. 4I). Consequently, cracks impinging upon the vein fiber are prone to be deflected along the film–fiber interface rather than penetrating the vein, hence exhibiting distinct fracture resistance in both radial and circumferential directions.

The radial vein design reduces structural mass and ensures that, even if cracks propagate along a vein, the wing retains sufficient aerodynamic area by simply separating into fan-like sectors. To compare flight performance between the intact and fractured seeds, we first dropped the intact seeds (*n* = 40) and the seeds with five (*n* = 40) or ten (*n* = 40) radial cracks from a height of 1 m in a windless environment (Fig. S11E). The horizontal distance from the release point to the landing point on the ground ranged from 5 cm to >160 cm for the intact seeds (Fig. 4J), with an average dispersal distance of 44±30 cm and an average falling velocity of 0.30 ± 0.05 m/s (Fig. 4J, K). Notably, the dispersal distance and the averaged falling velocity of the seed with five or ten radial cracks on the wing showed negligible differences compared to intact seeds (Fig. 4J, K), indicating that radial cracks have a trivial impact on the wing’s aerodynamic performance. Next, we circumferentially cut the intact seed wing to reduce its area to 2/3, 1/3, and 0 (bare core) of its original size (*n* = 40 for each group, Fig. S11E), and the average flight distance of the samples sharply decreased by 39%, 52%, and 73%, respectively, while the average falling velocity reached 0.4, 0.7, and 1.0 m/s, respectively (Fig. 4J, K). These results strongly suggest that the circumferential fracture patterns could be highly detrimental to wing performance. Building on these insights into stiffness and toughness, we further discuss how the geometrical parameters of the wing have been shaped to their current configuration in the Supplementary Materials (Fig. S12).

### Bioinspired filmy-wing carrier

**Figure 5.**
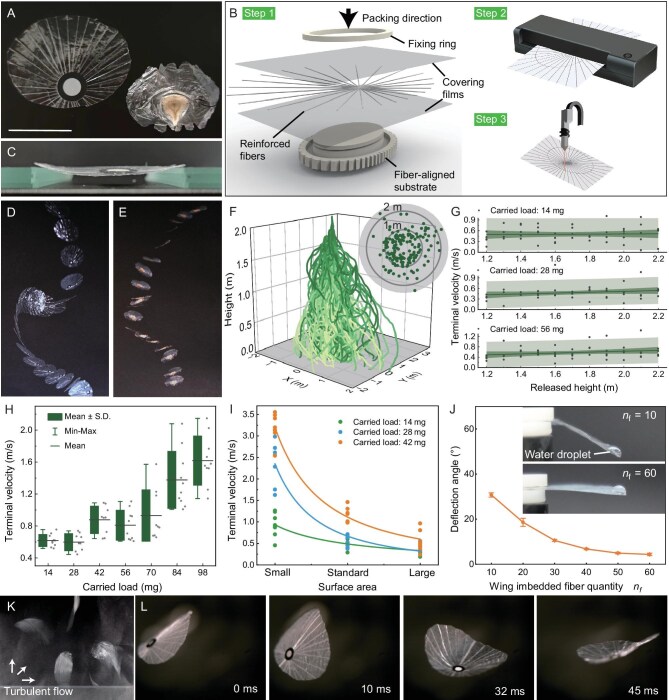
Passive micro-flier inspired by the seed. (A) Our fabricated micro-flier compared side by side with a natural seed. Scale bar, 2 cm. (B) Fabrication procedures of the bionic micro-flier including three steps: (1) packing the fibers into two covering films, (2) heat pressing to exclude the air, and (3) laser cutting for customizing shape and size. (C) Lateral view of the micro-flier supported by two side ends exhibiting insignificantly deformed wing when it carries a payload twice its own weight. (D) and (E) show the superposed high-speed frames of the freely falling process of the bionic micro-flier and the natural seed, respectively. (F) Superposed flight trajectories of the bionic micro-fliers carrying three typical loads, released from 1.2–2.2 m at an interval of 0.1 m in each drop test. Deeper green color indicates higher released height. Inset: radar graph depicting the maximum horizontal displacement of the micro-fliers during flight. (G) Terminal vertical velocity of the micro-fliers carrying three typical loads during the flight released from different heights. Dots and lines denote the experimental data and the linear fit, respectively. The deep and light green bands represent the confidence interval and the prediction interval, respectively. (H) Terminal velocity of the micro-fliers released from 2 m with different carried loads. (I) Terminal velocity of the loaded micro-fliers with three different surface areas, classified by small, standard, and large. Their wing spans are 20 mm, 40 mm, and 60 mm, and their flier weights are 7 mg, 21 mg, and 43 mg, respectively. Colored dots and curves represent experimental data and allometric fits. (J) Deflection angle of the micro-fliers with different quantities of the embedded fibers when loaded with an 8 μL water droplet. (K) Experimental setup for observing the micro-fliers in turbulent airflow conditions. (L) Selected frames from a high-speed video showing the fast-rotating process of the micro-flier in the turbulence.

Inspired by natural design strategies in which heterogeneous veins serve as basic building blocks that radiate to form seeds, we designed a passive micro-flier featuring vein-like structures arranged radially on an elliptical film (Fig. 5A). The micro-flier consisting of slender fibers embedded in two layers of thin film was fabricated through packing, heated-pressing, and laser cutting (Fig. 5B, Methods). Here, we used 80 μm-wide nylon fibers and 1 μm-thick polyethylene (PE) films to fabricate the micro-flier; both materials are widely available commercial products with excellent mechanical properties. Importantly, our micro-flier offers substantial design flexibility and can be constructed from various materials to satisfy specific requirements—for instance, biodegradable formulations (Fig. S13). Its size and the number of embedded fibers can also be customized (Fig. S14). The weight of the fliers, which incorporate 10 to 60 embedded fibers, ranges from 4.6 to 49.5 mg. When a 21 mg micro-flier (wing span 40 mm) was only supported by its two side ends and carried a payload twice its own weight, it was still able to retain an extended shape with most of the filmy wing suspended in the air, demonstrating the reliable stiffness of the fabricated wing (Fig. 5C). Furthermore, the free-falling trajectories of the micro-fliers exhibited a highly similar pattern to the natural seed (Fig. 5D, E, Videos S5, S6).

To quantify the air-dispersal performance of the micro-fliers with carried loads, free-falling tests were conducted in a windless indoor environment (Fig. 5F). The terminal vertical velocity of the flier showed insignificant variation when released at heights from 1.2–2.2 m, indicating that the micro-fliers can rapidly reach a stable falling state (Fig. 5F, G, Fig. S15). At a given release height, the addition of the payload monotonically increased the terminal velocity, confirming the critical impact of the micro-flier’s weight in its air-dispersal performance (Fig. 5H). Notably, even when the micro-flier carried a payload (56 mg) more than twice its own weight, the terminal falling velocity remained under 1 m/s, which enables a moderate breeze (1–5 m/s) to carry the flier to a distance exceeding its release height (Fig. 5H). Even with a payload of more than four times its own weight, the average terminal falling velocity could still be maintained at ∼1.5 m/s, thus demonstrating excellent load-bearing capacity.

When the payload is predetermined, as a given mission generally does, the falling velocity can be modulated by adjusting the size of the micro-flier. Increasing the surface area of the micro-flier could provide more aerodynamic forces and reduce its terminal velocity, although this reduction diminishes as the wing’s area is further expanded since the flier will eventually reach a minimum speed (Fig. 5I). The deformability of the bionic micro-flier can be fine-tuned by adjusting the number of embedded fibers (Fig. 5J). When tested in turbulent airflow, the bionic micro-flier exhibited highly random motion (Video S7), yet its wing structure remained intact after a 10-min wind-blowing test. Even when its rotating speed reached ∼10 r/s during flight in turbulence, the filmy wing showed insignificant deformation, demonstrating ideal aerodynamic adaptability (Fig. 5K, L).

**Figure 6.**
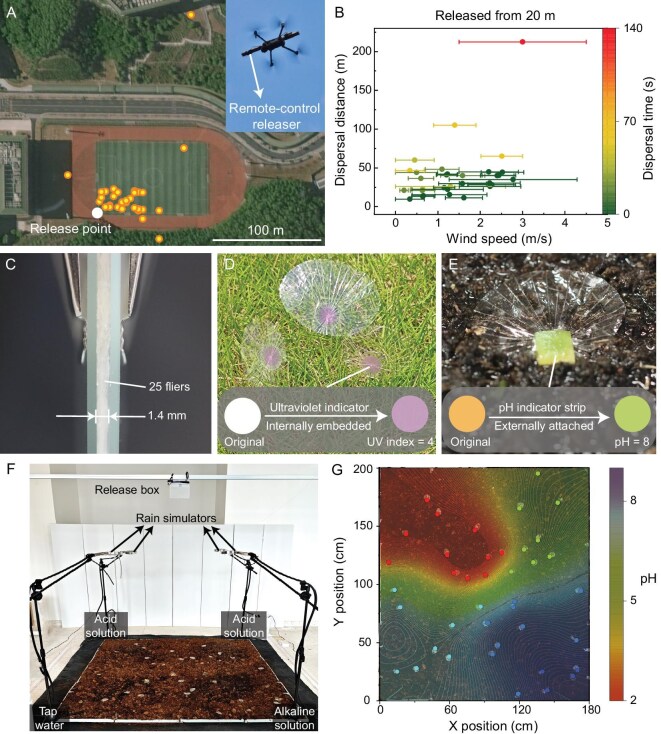
Field applications of the passive micro-flier. (A) Data points in the map showing the dispersal locations of the micro-fliers in the outdoor drop tests from 20 m height (*n* = 30). (B) Dispersal distances and the spent times of the micro-fliers in the drop tests shown in (A). (C) Twenty-five micro-fliers clamped between two glass slides with thickness of only 1.4 mm. (D) Micro-flier internally embedded with an indicator for ultraviolet detection. (E) Micro-flier externally attached with an indicator strip for soil pH detection. (F) Experimental setup showing the field application of the micro-fliers used for rain and soil detection. (G) Contour illustrating the pH distribution of the wet soil plotted according to the interpolation of the pH values detected by the micro-fliers.

The sufficient stiffness of the large wing area enables the lightweight micro-flier to stably disperse in the real-world scenarios. For instance, when we released our micro-fliers (wing weight: 40 mg, carried load: 40 mg, *n* = 30) in a light wind (wind speed: 0–4.5 m/s) from a 20 m height via a drone in an outdoor environment (Fig. 6A), most fliers traveled 10–105 m away from the point of release, except one sample dispersed to 212 m away from the origin, which may have experienced upwash wind during its flight. Apart from the exceptional sample, the average falling time of the other samples was 28 ± 16 s, suggesting an average falling velocity of 0.71 m/s (Fig. 6B). This falling speed should be low enough to allow the micro-flier to spread along with the wind, and can prevent damage if carrying electronic micro-devices [8]. In this practical experiment, we found most of the micro-fliers stayed intact after landing, except one sample which was damaged by the drone propeller. This sample exhibited a fractured pattern at the wing edge (Fig. S16), and the crack on the wing film could be terminated by the embedded fiber, resembling the function of the vein fiber of the natural seed.

We note that our flat micro-fliers are exceptionally suitable for large-scale air-delivery scenarios, as they can be stacked into a compact pile due to their exceptionally thin filmy wing, which greatly reduces the storage space in practical drone missions (Fig. 6C). Additionally, environmental indicators or other electronic devices that should avoid liquid contact can be embedded within the filmy wing for protection (Fig. 6D). Similar to natural seeds, the bionic wing, with its large surface area and thin profile, also exhibited good conformal attachment to wet soil, enabling it to detect soil properties when an indicator strip is externally attached to the wing (Fig. 6E).

To further exemplify the field detection capability of the micro-fliers, we selected pH as the environmental factor and evaluated a rectangular soil region after an artificial rain (see Methods). We first dropped 40 micro-fliers carrying pH colorimetric indicators from a height of 2 m, allowing random distribution of the micro-fliers on the soil substrate, and then initiated the artificial rain, with different solutions sprayed out from the rain simulators (Fig. 6F, Methods). The pH colorimetric indicators on the micro-fliers would immediately change their color upon being hydrated by the rain or the wet soil, and the corresponding pH values at every location could be easily captured by camera. Then, we were able to directly visualize the pH distribution of the entire region in a contour by interpolating the pH data points (Fig. 6G), which demonstrates an efficient and low-cost approach for monitoring environmental factors across a large field.

## DISCUSSION

Plants have evolved specialized structures and heterogeneous materials to achieve remarkable adaptation to complex environments [35,36]. In this study, we discovered that the seeds of the African tulip tree possess ultrathin filmy wings that are exceptionally lightweight, enabling slow descent and long-distance dispersal. The elastic modulus of the wing film is comparable to insect wing films, but its minimal thickness can be even smaller than that of dragonfly or cicada wings [37,38], which likely results from the seed’s lighter payload. The specialized veins covered by the stiff films can reinforce the wing structure, maintaining extension even in turbulent airflow to prevent folding and ensure stable flight. Unlike the hollow, mesh-like veins of insect wings [39], these radial veins are filled with soft, deformable fibers that can dissipate stress and resist crack propagation, enhancing toughness [29]. This combination of a ‘stiff but brittle’ film and ‘soft but tough’ fibers supports flight with minimal weight, while confining crack propagation to preserve aerodynamic function, which embodies an elegant natural solution that balances lightness, proper stiffness, and unidirectional toughness.

We emphasize that the wing structure should serve multiple functions throughout the seed’s life cycle. Although the seed’s aerial flight may last only minutes or hours, it must then remain on the soil for weeks before germination. Beyond enabling flight, the seed wing can conformably deform under capillary forces on uneven, wet substrates [40], increasing the contact area for attachment, water absorption, and nutrient uptake. Conversely, on dry soil, capillary forces are absent and gravity alone is insufficient to deform the wings; this allows the seed to potentially ‘sail off’ for secondary dispersal toward more favorable hydration conditions [3]. The innate ability to distinguish between wet and dry environments constitutes a form of physical intelligence, enabling the seed to ‘navigate’ to a suitable habitat [41,42]. In summary, the wing must reconcile the competing demands of rigidity for flight and flexibility for ground adhesion, which explains why the structure is not optimized solely for mechanical stiffness, but is instead a delicate compromise shaped by diverse environmental pressures, providing the seed with multi-functional adaptability.

In recent years, studies of wind-dispersed seeds have greatly inspired the development of passive micro-fliers, showing promising potentials in environmental monitoring, disease management, and other applications requiring coverage over expansive spatial ranges [7,8,18,43–47]. Our fabricated flier with a bio-inspired wing design holds many comparable advantages resembling the seed of the African tulip tree: (1) sufficient stiffness to stabilize the wing in random turbulence, (2) fracture-resistance in harsh environments, (3) conformability to attach to an uneven wet substrate for topsoil detection, and (4) stackability for large-scale dispersal in the air. Although our current artificial fliers are still inferior to the natural one in terms of descending velocity and mass density, the falling velocity of ∼0.60 m/s shown in our 35 –49 mg micro-fliers is significantly smaller than the descending speeds of existing micro-flier products at comparable weights, which were ∼1.0 m/s [18,46]. This improved flight performance is largely attributable to both the reduced weight and the large wing area that can provide substantial aerodynamic force.

Beyond replicating these natural features, our design offers unique practical advantages. Its aerodynamic performance can be flexibly tuned by tailoring the wing area and embedded fiber quantity to fit the payload weight and desired falling velocity. Furthermore, the wing can be customized with materials offering specialized properties such as high strength, water resistance, biodegradability, or stimuli-responsiveness to meet specific operational needs. Functional payloads, from electronic micro-devices to colorimetric indicators, can also be integrated either internally or externally, enhancing versatility in sensor packaging and protection. Overall, our bionic micro-flier exhibits excellent wing adaptability and strong load-carrying capacity for applications in practical scenarios, illuminating new possibilities in large-scale aerial delivery for wide-range sampling and environmental monitoring.

## METHODS AND MATERIALS

The main contents of the methods and materials of this work are presented in the Supplementary Materials.

### Wing structure model for deformation prediction

The Absolute Nodal Coordinate Formulation (ANCF) is a well-established method for analyzing large deformations in beam and plate structures [48,49]. We here combined our experimentally derived physical parameters with the ANCF method to analyze the bending deformation of the seed film when applied with different loads (Figs S8–S10).

The global position vector of an arbitrary point *P* on the neutral axis of the beam element can be written as


(1) \begin{eqnarray*}
{{\bf r}} = {\left[ {{r}_1,\ {r}_2,\ {r}_3} \right]}^T = {{\bf S}}\left( x \right){{\bf e}}.
\end{eqnarray*}

Here, ${{\bf e}}$ is the nodal coordinate vector and ${{\bf S}}( x )$ is the element shape function matrix, which forms are given in the Supplementary Data.

In order to develop the equations of deformation of the strip unit in the ANCF model, the vector ${{{\bf Q}}}_{\mathrm{k}}$ of the elastic forces and the vector ${{{\bf Q}}}_{\mathrm{e}}$ of the externally applied forces must be defined.

Axial strain energy of the beam element is defined as:


(2) \begin{eqnarray*}
{U}_l = \frac{1}{2}\mathop \int \nolimits_0^l {E}_l\varepsilon _l^2dx ,
\end{eqnarray*}

where ${U}_l$, ${\varepsilon }_l$, and *l* are axial strain energy, axial strain of the strip element, and initial length of the beam element, respectively. ${E}_l = {E}_f( {\pi ( {{D}^2 - {d}^2} )/4 + {b}_f \cdot {h}_f} ) + \pi {E}_{in}{d}^2/4$ is the effective tensile stiffness;

${\varepsilon }_l$ is defined using the Green–Lagrange strain tensor and can be written as


(3) \begin{eqnarray*}
{\varepsilon }_l &=& \frac{1}{2}\left( {{f}^2 - 1} \right) = \frac{1}{2}\left( {{{{\bf r}}}^{\mathrm{T}}{{\bf r^{\prime}}} - 1} \right)\\
&=& \frac{1}{2}\left( {{{{\bf e}}}^T{{{{\bf S^{\prime}}}}}^T{{\bf S^{\prime}e}} - 1} \right),\quad{\mathrm{\ }}{{\bf S^{\prime}}} = \frac{{{\mathrm{d}}{{\bf S}}}}{{{\mathrm{d}}x}}.
\end{eqnarray*}

Axial elastic force vector ${{{\bf Q}}}_l$ is derived by differentiating ${U}_l$ with respect to ${{\bf e}}$


(4) \begin{eqnarray*}
{{{\bf Q}}}_l = {\left( {\frac{{\partial {U}_l}}{{\partial {{\bf e}}}}} \right)}^{\mathrm{T}} = {{{\bf K}}}_l{{\bf e}}.
\end{eqnarray*}

where ${{{\bf K}}}_l = {E}_l\overline {{\varepsilon }_l} \mathop \smallint \nolimits_0^l ( {{{{{\bf S^{\prime}}}}}^T{{\bf S^{\prime}}}} ){\mathrm{d}}x$ is the nonlinear axial stiffness matrix and $\overline {{\varepsilon }_l} $ is the mean axial strain and can be calculated as $\overline {{\varepsilon }_l} = ( {{l}_s - l} )/l$, and


(5) \begin{eqnarray*}
{l}_{\mathrm{s}} = \mathop \int \nolimits_l {\mathrm{d}}s = \mathop \int \nolimits_0^l \sqrt {{\boldsymbol{r}}_x^{\mathrm{T}}{{\boldsymbol{r}}}_{\mathrm{x}}} {\mathrm{d}}x = \mathop \int \nolimits_0^l \sqrt {{{\boldsymbol{e}}}^{\mathrm{T}}{{\bf S}}{^{\prime}}^T{{\bf S^{\prime}}}{\boldsymbol{e}}} {\mathrm{d}}x .
\end{eqnarray*}

Set $f( x ) = {{\boldsymbol{e}}}^{\mathrm{T}}{{\bf S}}{^{\prime}}^T{{\bf S^{\prime}}}{\boldsymbol{e}} - 1$, where ${{\bf S^{\prime}}}$ is the derivative of ${{\bf S}}$ with respect to *x*, then ${l}_{\mathrm{s}}$ can be written as


(6) \begin{eqnarray*}
{l}_{\mathrm{s}} = \mathop \int \nolimits_0^l \sqrt {f\left( x \right) + 1} {\mathrm{d}}x.
\end{eqnarray*}

By expanding the term $\sqrt {f( x ) + 1} = 1 + \frac{{f( x )}}{2} - \frac{{f{{(x)}}^2}}{8} + \cdots $, and adopting the first two terms we get


(7) \begin{eqnarray*}
{l}_{\mathrm{s}} = \& \mathop \int \nolimits_0^l \sqrt {f\left( x \right) + 1} {\mathrm{d}}x \cong \frac{1}{2}\left[ {{{\boldsymbol{e}}}^{\mathrm{T}}\bar {{{{\bf S}}}_{\boldsymbol{l}}} {\boldsymbol{e}} + l} \right].
\end{eqnarray*}

where $\bar {{{{\bf S}}}_{\boldsymbol{l}}} = \mathop \int \nolimits_0^l {{\bf S}}{^{\prime}}^T{{\bf S^{\prime}}}{\mathrm{d}}x$_._ Then $\bar {{\varepsilon }_l} = ( {{{\boldsymbol{e}}}^{\mathrm{T}}\bar {{{{\bf S}}}_{\boldsymbol{l}}} {\boldsymbol{e}} - l} )/2l$. Finally, we have


(8) \begin{eqnarray*}
{{{\bf K}}}_l = {E}_l\bar {{\varepsilon }_l} \mathop \int \nolimits_0^l {{{\bf S^{\prime}}}}^T{{\bf S^{\prime}}}{\mathrm{d}}x = \frac{{{E}_l}}{{2l}}\left( {{{{\bf e}}}^{\mathrm{T}}\bar {{{{\bf S}}}_{\boldsymbol{l}}} {{\bf e}} - l} \right)\bar {{{{\bf S}}}_{\boldsymbol{l}}}.
\end{eqnarray*}

Next, the bending strain energy of the strip element is defined as


(9) \begin{eqnarray*}
{U}_t = \frac{1}{2}\mathop \int \nolimits_0^l B{\kappa }^2dx.
\end{eqnarray*}

where ${U}_t$, *B*, and $\kappa $ are bending strain energy, bending stiffness, and curvature, respectively.

The curvature can be calculated as


(10) \begin{eqnarray*}
\kappa = \left| {\frac{{{{\mathrm{d}}}^2{{\bf r}}}}{{{\mathrm{d}}{s}^2}}} \right|,\quad\ {\kappa }^2 = {{{\bf e}}}^{\mathrm{T}}{{\bf S^{\prime\prime}S^{\prime\prime}e}}.
\end{eqnarray*}

The bending strain energy then writes


(11) \begin{eqnarray*}
{U}_t &=& \frac{1}{2}\mathop \int \nolimits_0^l B{\kappa }^2{\mathrm{d}}x\\
&=& \frac{1}{2}\mathop \int \nolimits_0^l B{{{\bf e}}}^{\mathrm{T}}{{\bf S^{\prime\prime}S^{\prime\prime}e}}{\mathrm{d}}x,{\mathrm{\ }}{{\bf S^{\prime\prime}}} = {{\bf S^{\prime}}}/dx .
\end{eqnarray*}

Bending elastic force vector ${{{\bf Q}}}_t$ can be written as the product of the bending stiffness matrix ${{{\bf K}}}_t$ and the nodal coordinate vector:


(12) \begin{eqnarray*}
{{{\bf Q}}}_t = {\left( {\frac{{\partial {U}_t}}{{\partial {{\bf e}}}}} \right)}^{\mathrm{T}} = \left[ {\mathop \int \nolimits_0^l B{{{\bf S}}}^{{\mathrm{^{\prime\prime}T}}}{{\bf S^{\prime\prime}}}{\mathrm{d}}x} \right]\ {{\bf e}} = {{{\bf K}}}_t{{\bf e}}.
\end{eqnarray*}

Hence, the bending stiffness matrix is in the form:


(13) \begin{eqnarray*}
{{{\bf K}}}_t = \mathop \int \nolimits_0^l B{{{\bf S}}}^{{\mathrm{^{\prime\prime}T}}}{{\bf S^{\prime\prime}}}{\mathrm{d}}x.
\end{eqnarray*}

External force vector ${{{\bf Q}}}_{\mathrm{e}}$ is defined using the virtual work as


(14) \begin{eqnarray*}
{\mathrm{\delta }}{W}_e = \delta {{{\bf F}}}^T\delta {{\bf r}} = {{{\bf F}}}^T{{\bf S}}\delta {{\bf e}} = {{\bf Q}}_e^T\delta {{\bf e}}.
\end{eqnarray*}

For a uniformly distributed pressure *q* (${\mathrm{N}}/{{\mathrm{m}}}^2$) applied on the element, the virtual work can be written as:


(15) \begin{eqnarray*}
{\mathrm{\delta }}{W}_e = \mathop \int \nolimits_0^l qb\ \delta {{\bf r}}{\mathrm{d}}x = \mathop \int \nolimits_0^l qb\ {{\bf S}}\ {\mathrm{d}}x\ \delta {{\bf e}}.
\end{eqnarray*}

which yields ${{{\bf Q}}}_{\mathrm{e}} = qb\mathop \int \nolimits_0^l {{{\bf S}}}^T{\mathrm{d}}x$

Finally, the static equation of the strip can be obtained with the bending and axial stiffness matrices and external force vector as


(16) \begin{eqnarray*}
{{{\bf K}}}_t{{\bf e}} + {{{\bf K}}}_l{{\bf e}} = {{{\bf Q}}}_{\mathrm{e}}.
\end{eqnarray*}

The beam is set with one end fixed (all the degrees of freedom are set to be zero) and the other end free. The static equation of the beam is nonlinear with respect to ${{\bf e}}$ which can be solved via the Newton–Raphson method. The validity of our model is verified by comparing the predicted deformation profile to the finite element analysis result (Fig. S10).

## Supplementary Material

nwag132_Supplemental_Files
