# Supplementary material for: Ultrathin seed wing with heterogeneous structures for highly efficient dispersal of African tulip tree
Source: Natl Sci Rev. 2026 Mar 3;13(9):nwag132. doi: 10.1093/nsr/nwag132 (PMC13189003; doi:10.1093/nsr/nwag132)
Supplement: nwag132_Supplemental_Files [file nwag132_supplemental_files.zip › Supplementary Data.pdf]

# SUPPLEMENTARY DATA for

## Ultrathin seed wing with heterogeneous structures for highly efficient dispersal of African tulip tree

Jiangkun Wei[1,#], Mingshen Wu[2,#], Yongtao Dai[1], Peiyu Cao[1], Hao Yang[1], Zheng Chen[1], Jinzhao Yang[1], Zhigang Wu[1,*], Stanislav Gorb[3,*] and Jianing Wu[1,2,*]

[1]School of Aeronautics and Astronautics, Sun Yat-Sen University, Shenzhen 518107, China;

[2]School of Advanced Manufacturing, Sun Yat-Sen University, Shenzhen, 518107, China;

[3]Functional Morphology and Biomechanics, Zoology Department, Kiel University, Kiel 24118, Germany

[*]**Corresponding authors.** E-mails: wuzhigang@mail.sysu.edu.cn; sgorb@zoologie.uni-kiel.de; wujn27@mail.sysu.edu.cn

[#] Equally contributed to this work.

**The file includes:**

Materials and methods

Supplementary information (including figures S1 to S16)

Legends for videos S1 to S7

References

**Other Supplementary Materials for this manuscript include the following:**

Videos S1 to S7

# MATERIALS AND METHODS

## Flight performance characterization

The seeds of African tulip tree were sourced from outdoor trees planted at Guangzhou campus and Shenzhen campus of Sun Yat-Sen University, China (23°N, 113°E). The mature seeds of the African tulip tree were collected from natural seed pods (fig. S1). To observe how the seed wing deforms in the turbulent airflow, we deposited the seed samples ($n$=100) in a glass box (size: $38 \times 26 \times 32$ cm) and blew the samples via an air compressor. The flow speed at the front opening of the blower reached 5 m/s. As soon as we switched on the air compressor, the seeds inside the box flew in a fast and highly random pattern, and the motion of the seed was recorded at 1000 frames per second (fps) by a high-speed camera (Phantom, VEO E, USA). To characterize the deformation of the seed wing, we measured the time-varying rotation angle of the seed core and the deflection angle of the seed wing, respectively, when the seed rotated in the turbulence. The flight trajectory of the seed in the calm air was recorded by the high-speed camera ($n$ = 20).

## Adhesion and deformability test

To study the deformability of the seed on wet soil, six substrates with different sinusoidal curvature surfaces were designed and printed by a stereolithography 3D printer (Formlabs Form 3, USA) to represent the natural soil substrate fluctuation. Each substrate had a constant wavelength $\lambda$ of 5 mm but with different amplitude $A$ at 0.5, 0.6, 0.7, 0.8, 0.9, and 1 mm, respectively. The seed ($n$ = 64) was gently placed on the substrate pre-wetted by 1% wt/wt sucrose solution and then desiccated overnight under indoor conditions. We defined the lateral side where we could observe the sinusoidal curve of the surface as an 'observation plane'. In the context, we placed the seeds on the substrates in two directions: (1) depth direction: the symmetrical center line of the seed core perpendicular to the observation plane, and (2) sinusoidal direction: the symmetrical center line of the seed core parallel to the sinusoidal curve direction. Then, averaged curvature of the wing film in one sinusoidal wavelength was measured from the view of observation plane using Fiji (segment line module and Kappa module, version 1.53t).

## Characterization of microstructure and material compositions

Scanning electron microscopy (SEM) was used to examine the microstructure of the seed wing ($n$ = 20). Intact seeds were collected from seed pods and were observed at different magnifications under SEM. We also manually stretched the wing to randomly add some cracks on it to observe how the wing microstructure reacted to the crack. Specimens were sputter-coated by a Platinum film and then imaged with an SEM (MAGNA, Tescan, Czech) at 5 kV. We also recorded the EDS spectrum and the element distribution of the seed wing under area scan and line scan modes, respectively.

To acquire more material information about the seed wing, wing samples ($n$ = 10) were stained and visualized with a confocal laser scanning microscopy (CLSM) instrument (LSM 880, Carl Zeiss Microscopy, Germany). The cell walls in plants are made up of cellulose, hemicellulose, lignin and so forth [1]. We used 0.2% Basic Fuchsin (Rhawn, China), 0.1% Calcofluor White (Coolaber, China) and 0.05% Nile Red (Coolaber, China) in ClearSee solution (Wako, Japan) to examine the existence of lignin, cellulose and suberin, respectively. Sample processing and imaging procedures follow strictly to the corresponding protocols [2].

## Characterization of mechanical properties

To measure the elastic moduli of the wing film and the vein fiber, we amputated the seed wing to expose the inner fiber and glued the sample on a glass slide. The specimens were indented in air environment (24°C) using an atomic force microscopy (AFM) instrument (Bruker, Dimension Fast scan Bio, Germany) equipped with a probe RTESP-300 (spring constant: 5 N/m). To ensure that the effects of the substrate are insignificant, the indentation depth was set at 15 nm, being less than 5% of the tested sample thickness.

According to the classical Griffith's fracture theory, the material toughness can be characterized by energy release rate $\Gamma$, which measures the crack energy consumption per increased fracture surface area. We used a digital force gauge (Force Sensor Capacity: 0.5 N and 10 N, Mark-10, USA) with a sampling rate of 250 Hz and a resolution of 0.02 N to conduct the force measurements. The wing films were carefully amputated into 8 mm width samples (sample size: $n$ = 13). We examined the fracture energy of the samples in two directions: (1) when the tension direction is parallel to the strip direction ($n$ = 8), and (2) when the tension direction is perpendicular to the strip direction ($n$ = 5). Tensile tests were performed at a

displacement rate of 1 mm/min until the sample was fully separated, and load curves were obtained and integrated against the displacement to obtain fracture energy $W$ at each direction.

The toughness of the film can be characterized by its energy release rate $\Gamma_f = W/l_c h$, where $l_c$ is the crack length and $h$ is the thickness of the film. With the knowledge of $\Gamma_f$, the energy release rate of the inner fiber can then be extracted from the parallel tensile fraction test. The crack in the parallel tensile test is tortuous but can be viewed as the sum of all the longitudinal and transverse crack segments. In the parallel test, cracks randomly went up and down, and the length of each crack segment $i$ (before turning its longitudinal direction) projected on the transverse and longitudinal direction can be labelled as $l_t(i)$ and $l_l(i)$, respectively. All the longitudinal crack segments only separate the brittle film and the energy consumption for these longitudinal segments is $W_l = \Gamma_f h \sum l_l(i)$. The fracture energy of all the transverse crack segments can then be extracted from the total energy consumption, which writes $W_t = W - \Gamma_f h \sum l_l(i)$. The number of strips that the crack crossed through is $n = \sum l_t(i)/b$. In the transverse section of one unit strip (fig. 3J), the area composed by the film is the sum of the side film area $A_1 = h(b - h - d)$, the vein covering film area $A_2 = \pi((d+h)^2 - d^2)/4$, and the fiber area $A_3 = \pi d^2/4$. Hence, the energy required to separate all the film in transverse direction writes $W_f = n(A_1 + A_2)\Gamma_f$, by subtracting which we can obtain the energy required to separate the fibers $W_{in} = W_t - W_f$. In the context, the toughness of the fiber can be estimated as $\Gamma_{in} = W_{in}/nA_3$.

## Bionic passive micro-flier design and performance test

We designed the passive aerial micro-flier in an elliptical shape resembling the real seed (fig. 5A). We select low-cost, light-weight and high-strength artificial materials for fabricating the micro-flier. The commercial Polyethylene (PE) film was adopted due to its thin structure (thickness: 1 μm) and light-weight (density: 0.9 g/cm$^3$) (Young's modulus: 400 MPa). The nylon fishing line with a small diameter (80 μm), light-weight (density: 1.15 g/cm$^3$), and high stiffness (Young's modulus: 2 GPa) was also selected to reinforce the wing. In the flier, nylon fibers were embedded by two layers of PE films and were radially distributed with the density adjustable. The fiber intersection point was located at the symmetric axis of the flier and is 1/6 length of the minor axis away from the peripheral edge. We designed a packing device to facilitate aligning the fibers with a defined angle and embedding the fiber inside the PE films (fig. S13). The packed film need

to be heated-pressed by a hot rolling machine to expel the internal air and to firmly fix the fibers. The shape and the size of the micro-flier can be customized through laser cutting. We defined and fabricated ‘small’, ‘standard’, and ‘large’ micro-fliers with corresponding spans of 20, 40, and 60 mm and heights of 15, 30, and 45 mm for the performance test (fig. S14). Other degradable materials can also be adopted for the micro-flier as the fabrication process is highly flexible for diverse materials.

The flight performance of the micro-flier was mainly quantified by the maximum dispersal distance and the terminal falling velocity. We used an Optical Motion Capture System (OMCS, NOKOV, China) consisting of 12 high-resolution cameras to examine the flight performance of the bionic micro-flier. Two sizes of reflective stickers with the weight of 7 mg and 20 mg, respectively, were adopted as the addible payloads and as identification points for the OMCS. We tested the micro-fliers carried with three typical loads (14, 28, and 56 mg) during the flight at different released heights from 1.2m to 2.2 m, with an interval of 0.1 m in the windless indoor environment. Moreover, we also tested the micro-fliers that were released from 2 m with different payloads (14, 28, 42, 56, 70, 84, and 98mg). At least 5 flier samples were tested for each of the above sets. The flight performance of the micro-flier with different sizes (with the same embedded fiber number) was also examined. To observe how the micro-flier morphs in the turbulent airflow, we deposited the fliers ($n$ = 30) in the glass box and blew the samples via the air blower. The highly random motion of the flier in the turbulence was recorded at 1000 fps by the high-speed camera. To quantify the stiffness variation with the number of embedded fibers, we clamped one edge of the micro-flier and placed an 8 μL water droplet on the surface in other edge. The deflection angles were measured with respect to the different fiber numbers.

## Field applications of the passive micro-flier

To demonstrate the outdoor dispersal performance of our designed passive micro-fliers, we conducted the drop tests from 20 m at an open field. The experiments were conducted on a light-wind day with most of the wind speeds ranging from 0 to 3 m/s, and with the highest wind speed at about 4.5 m/s. The micro-fliers were first clamped by a remote-control releaser equipped on a drone (DJI Air 3, China) and were carried to 20 m height. We then dropped the micro-fliers as soon as any wind had passed by and the wind speed was recorded by an anemometer. For precisely tracking the dispersal location of the micro-fliers, we released two

fliers in each drop test, recorded the spent time upon falling and measured their relative positions with respective to the origin.

To exemplify the environmental detection capability of the micro-fliers, we set pH as a detection factor for the rain and soil evaluation. Commercial plant soils (Pindstrup, Denmark) were placed on a rectangular region with 180 cm in width and 200 cm in length. The soil substrate was uneven, with thickness ranged from 5 cm to 15 cm. Four sprinklers connected to four buckets were placed at each corner of the rectangular region to simulate the rain condition. The buckets contained tap water, alkaline solution ($NaHCO_3$), concentrated and diluted acid solution ($CH_3COOH$), respectively, to modulate the rain pH. The experiment began first by releasing forty micro-fliers carried with pH test strips via a remote-control drop box from 2 m. Upon the micro-fliers freely landed on the soil substrate, we switched on the sprinklers for 1 minute to allow the fliers to detect the pH of the rain or the wet soil. The pH values were then measured for the micro-fliers by colorimetric indicator, along with their positions. Finally, the pH distribution contour can be plotted by interpolating the pH data on the tested region.

# SUPPLEMENTARY INFORMATION

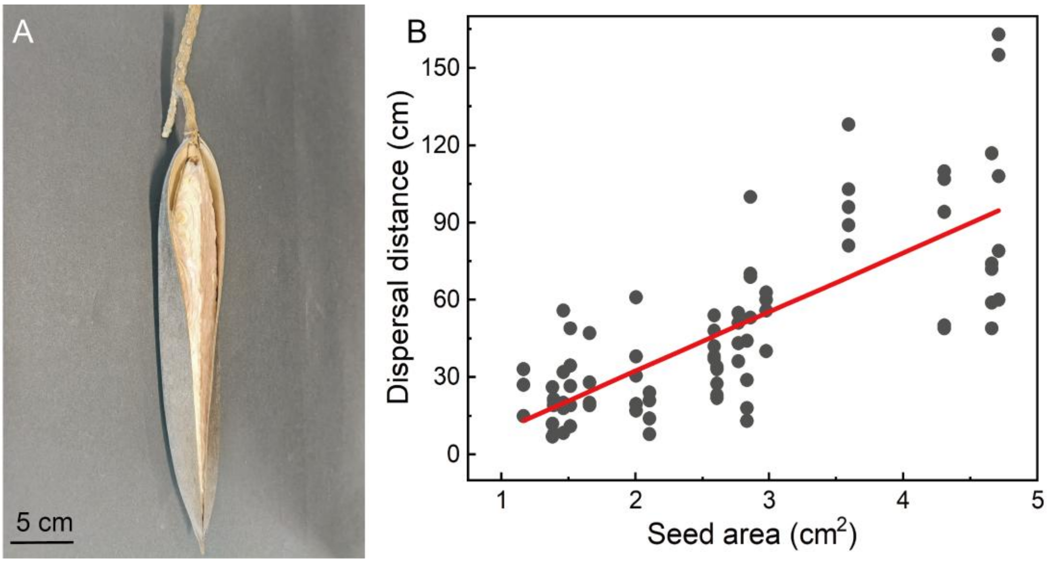


**Figure S1**. (A) Seed pod of the African tulip tree. (B) Seed dispersal distance with respect to different surface areas of the seed. Intact seeds ($n$=20) with different sizes were selected from the seed pod to examine how the wing area affects the seed dispersal performance. The area of the wing was first determined and then the seeds were released at the height of 1 m in a windless environment. The horizontal distances from the point of the release to the individual landed seeds were measured, with each seed tested five times.

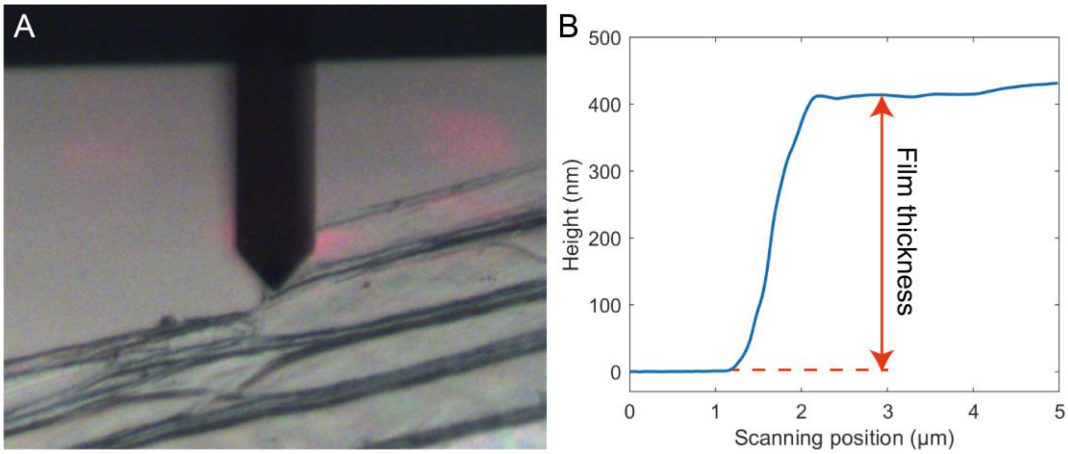


**Figure S2**. Examination of the wing film thickness. (A) The cantilever probe of the atomic force microscope (AFM) scanning the fractured wing film affixed to a glass slide. (B) Height profile data from the AFM scan, indicating that the wing film thickness, is measured at 414±13 nm (*n*=20). The 0 point was set at the glass substrate plane.

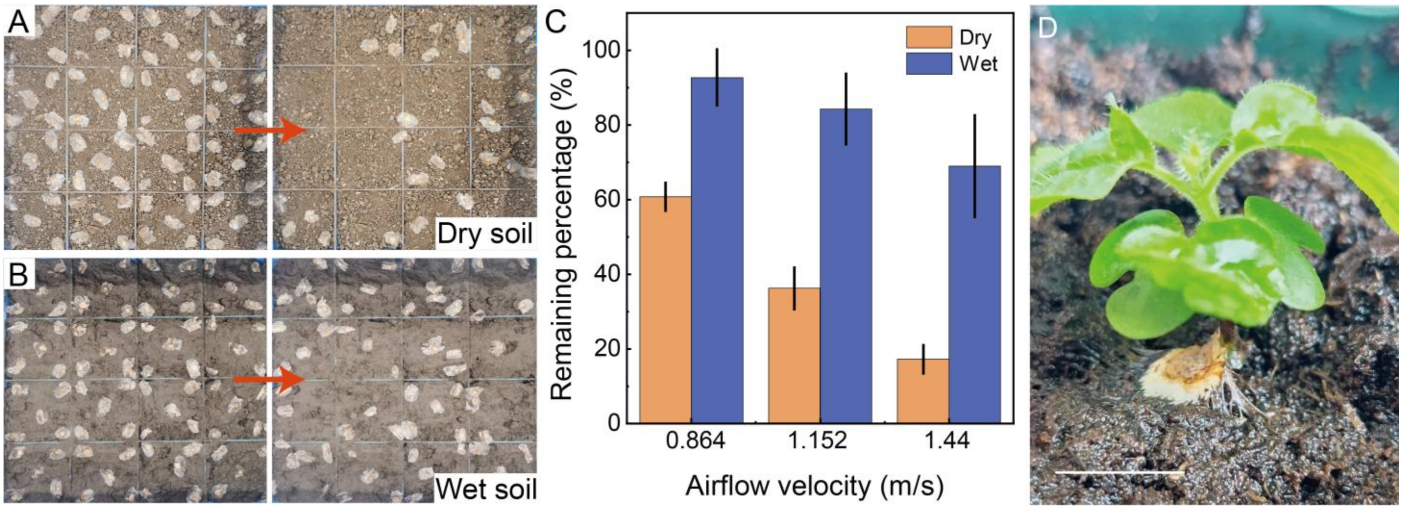


**Figure S3**. Assessment of seed adhesion to different soil conditions. (A) Seeds placed on dry (upper images) and (B) wet (lower images) soils (*n* = 80), respectively, which were kept within two 40 × 40 cm square boxes. A fan was used to generate horizontal wind from one side of the soil surface until all the seeds ceased to move. The fan could produce three wind speeds at 0.86, 1.15, and 1.44 m/s. Each wind speed was tested in at least three independent runs. (C) Percentage of the seeds remaining on the soil after airflow test. Yellow and blue columns represent seeds on the dry or the wet soils, respectively. On dry soil, 20%-60% of seeds remained, while on pre-wetted soil, 70%-95% of seeds stayed in place. The promotion of the percentage proportion indicates capillary force should dominate the seed adhesion when the soil is wet. (D) Seed wing remaining attached to the soil, stabilizing the stem after seed germination. Scale bar, 10 mm.

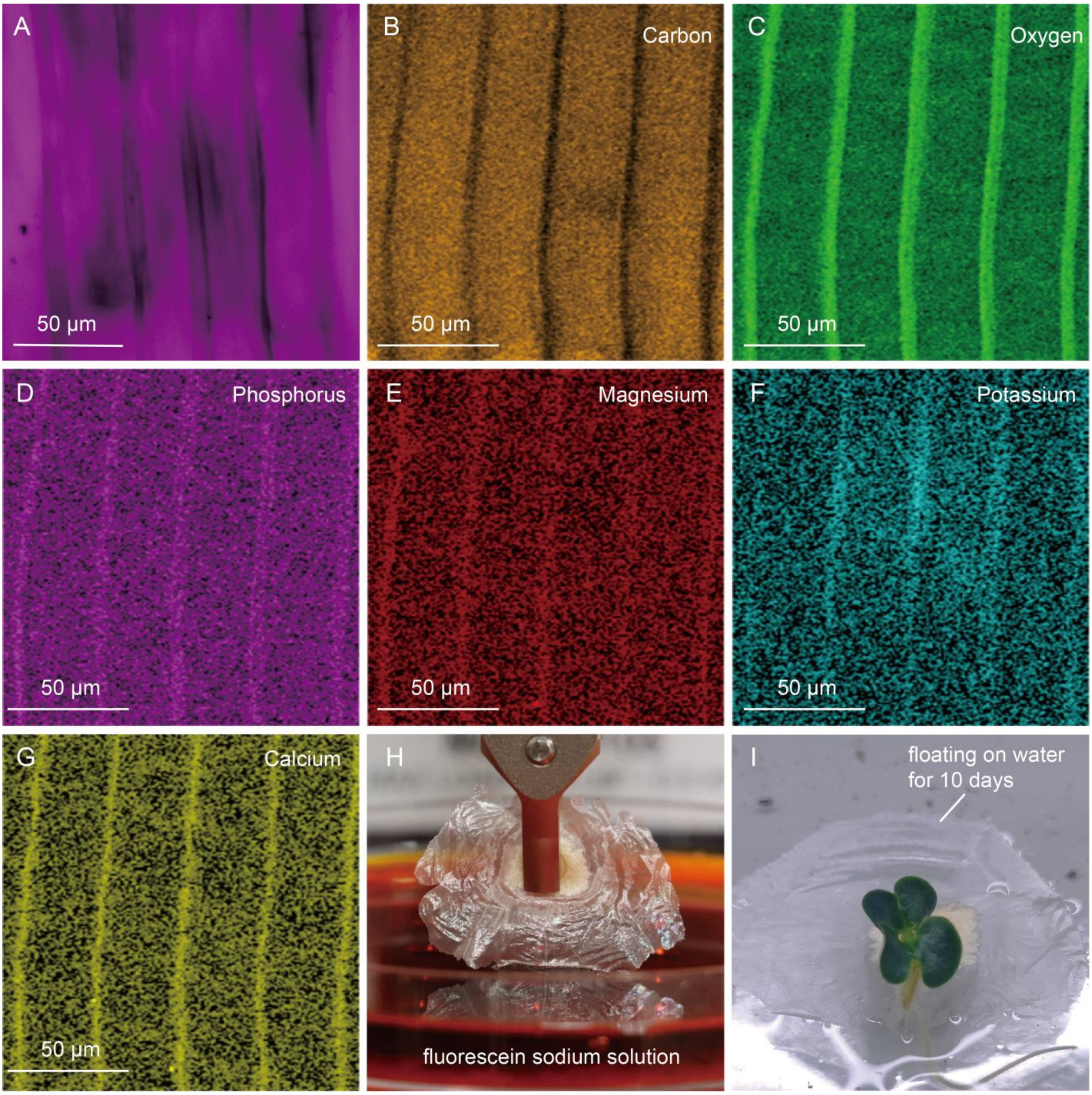


**Figure S4**. (A) CLSM image showing magenta fluorescence from the Nile Red-stained seed wing region, indicating the presence of suberin in both the wing film and the vein fibers[2]. EDS elements detection revealing the existence of (B) Carbon, (C) Oxygen, (D) Phosphorus, (E) Magnesium, (F) Potassium, (G) Calcium. (H) Seed wing immersed in a fluorescein sodium salt solution (Sigma, USA) for 24 hours (*n*=5) and examined using confocal laser scanning microscopy (CLSM, LSM 880, Carl Zeiss Microscopy, Germany) with a laser excitation wavelength at 488 nm. No fluorescein signal can be found under the CLSM, indicating no capillary effect of the vein-like structure and no hygroscopic effect of the wing film, and suggesting its pure mechanical function. (I) Seed wing remained extended after the seed floating on the water surface for 10 days until germination.

## Applied load in natural situation

### Aerodynamic force

We used computational fluid dynamics simulation (CFD) to evaluate the aerodynamic forces acting on the flying seed. Based on the seed's geometry, we simplified the seed model as an elliptical thin plate, with a thickness of 0.1 mm, a span of $l_{span} = 20$ mm and a height of $l_{height} = 12$ mm. To represent the seed core, we added a thickened circular plate (radius: 3 mm, thickness: 0.6 mm) tangent to the bottom edge of the elliptical plate. The *k-ω* turbulence model was employed to capture effects of turbulence. The fluid domain was set as a cylindrical computational region with 100 mm in cross-sectional radius and 200 mm in length. All the boundaries were more than 15 chord lengths away from the central object, which is sufficiently large to suppress the boundary effects. The flow velocity at the inlet boundary was set as 1 m/s and the pressure at the flow outlet was set as 0 Pa. Symmetrical boundary condition was assigned to other boundaries of the fluid domain and no-slip boundary condition was assigned to the surfaces of the seed object.

For the analysis of the static structure in the flow, the seed object was fixed at the center of the fluid domain and tested at different angles of attack $\alpha$ (the angle between the oncoming flow and the symmetric line of the seed plane) from $5°$ to $30°$ at the interval of $5°$ and from $30°$ to $90°$ at the interval of $10°$. The object surface was added with ten layers of inflation meshes (first layer size: 0.2 mm) and other flow space was discretized by a set of refined tetrahedral mesh to ensure computational accuracy. We recorded the drag coefficient $C_l = 2F_d/\rho u^2 A_{seed}$, the lift coefficient $C_l = 2F_l/\rho u^2 A_{seed}$ and the pressure $p$ on the seed model, respectively, where $F_d$ is the drag force, $F_l$ the lift force, $\rho_{air} = 1.225\ kg/m^3$ the density of the air, and $A_{seed} \cong 5\ \text{cm}^2$ the surface area[3].

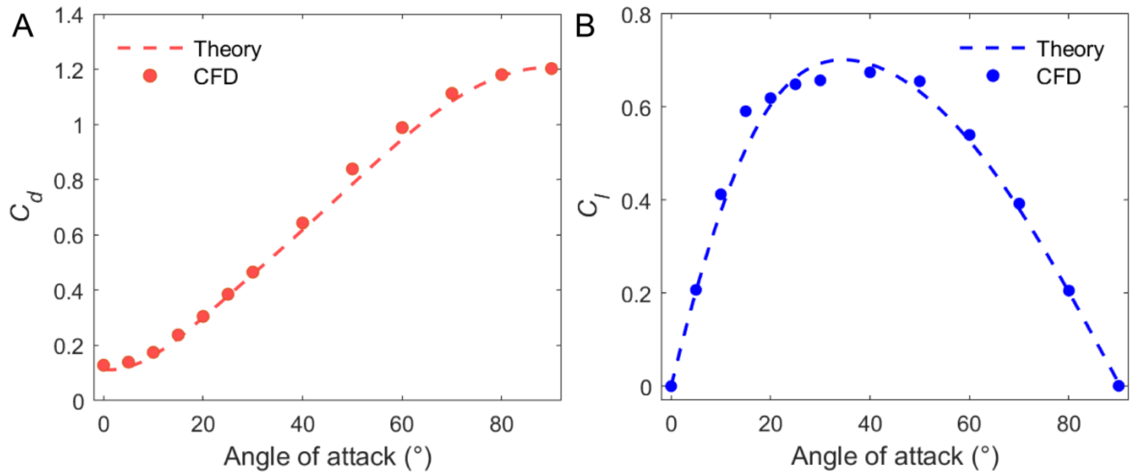


**Figure S5**. (A) Drag coefficient $C_d$ and (B) lift coefficient $C_l$ of the CFD seed model with a variation of the angle of attack.

The calculated drag and lift coefficient are shown in fig. S5 and can be explicitly expressed in the following forms[4]:

$$C_L(\alpha) = \tilde{f}(\alpha) \cdot C_L^1 \sin\alpha + \left[1 - \tilde{f}(\alpha)\right] \cdot C_L^2 \sin 2\alpha\,, \qquad \alpha \in [0, \pi/2]$$

and

$$C_d(\alpha) = \tilde{f}(\alpha) \cdot (C_D^0 + C_D^1 \sin^2\alpha) + \left[1 - \tilde{f}(\alpha)\right] \cdot C_D^{\pi/2} \sin^2\alpha\,, \qquad \alpha \in [0, \pi/2]$$

respectively, where

$$\tilde{f}(\alpha) = \frac{1 - \tanh\left[\dfrac{(\alpha - \alpha_0)}{\delta}\right]}{2}, \qquad \alpha \in [0, \pi/2]$$

The parameter values $C_L^1 = 2.95$, $C_L^2 = 0.55$, $C_D^0 = 0.15$, $C_D^1 = 3.5$ and $C_D^{\pi/2} = 1.2$ are chosen to yield good agreement with the measurements. The function $\tilde{f}(\alpha)$ is used to select either the laminar or separated (stalled) regime, where $\alpha_0 = 14°$ is the critical angle of attack at stall and $\delta = 25°$ determines the smoothness of the transition.

Previous study shows that the torque on a freely falling plate is one to two orders of magnitude smaller than the torque on a glider with fixed angle of attack, and the former situation is more suitable for the flying seed[5]. In the absence of translation motion, the torque term for a rotating seed in the air writes:

$$\Gamma = \frac{1}{64}\rho_{air}\, C_d^{\frac{\pi}{2}}\, l_{width} l_{span}^4 \dot{\theta}^2$$

where $\dot{\theta}$ is the angular velocity of the rotating seed. Then we have

$$P_{aero} \cong \frac{\tau}{l_{span}A_{seed}} = 2.9\ \mathrm{mPa}, \qquad \text{when } \dot{\theta} = 10\ \mathrm{r/s}$$

$$P_{aero} \cong \frac{\tau}{l_{span} A_{seed}} = 46.2 \text{ mPa}, \qquad \text{when } \dot{\theta} = 40 \text{ r/s}$$

**Capillary force**

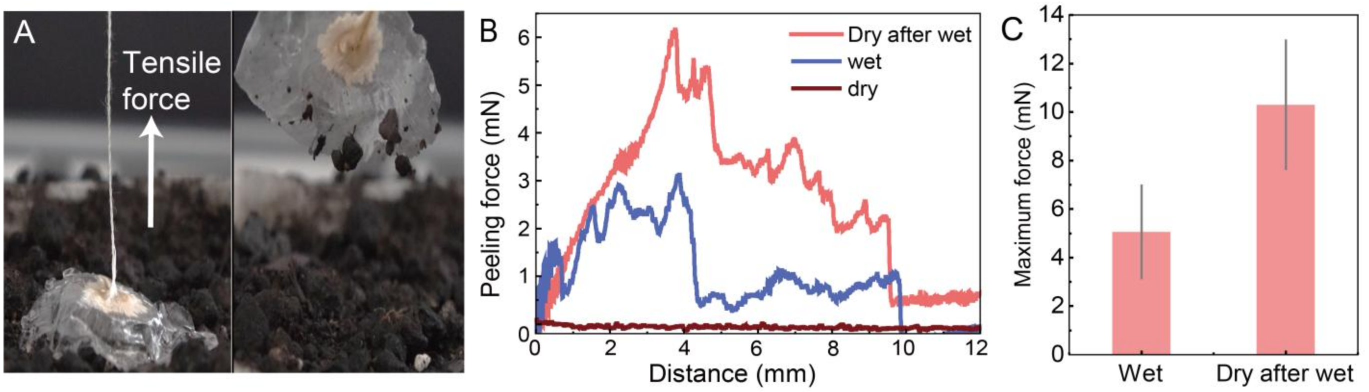


**Figure S6**. (A) Typical process of a seed peeling from the soil substrate. The forces of the seed peeling from the dry soil, the wet soil and the initially wet but eventually desiccated soil were recorded, respectively. (B) Experimental data of distance-related force of the seed peeling from the soil substrate ($n$ = 15). (C) The maximum force measured in the peeling test.

To estimate the capillary pressure, the pull-off forces of the seed peeling from the wet soil were recorded. The seed core was connected to a thread that linked to the force gauge of a motorized force tester (Series F, Mark 10, USA) for testing. For comparison, the pull-off forces of the seed peeling from the dry soil and the initially wet, but eventually desiccated soil, were also recorded. The overall and maximum pull-off forces of the seed placed on the wet soil surface were significantly larger than the forces of the seed placed on the dry soil (fig. S6A, B). The magnitude of the capillary pressure derived by the peeling test can be estimated by $F_p/A_{seed} \cong 10$ Pa, where $F_p \approx 5$ mN is the maximum peeling force from the wet soil. We also found in the experiment that, if the seed was initially placed on the wet soil and was desiccated overnight, the pull-off force would be even larger than the case on wet soil (fig. S6C), which reached ~10 mN, being 200 times larger than the weight of the seed itself. This may result from stronger conformal attachment between the seed wing and the soil particles as the initial connecting liquid bridge dried up, which is to some extent similar to adhesive performance of cellulose-containing mucilagenous seeds[6,7].

The capillary force applied by liquid can be described by the Young-Laplace equation, which states

$$\Delta p = -\gamma \left( \frac{1}{R_1} + \frac{1}{R_2} \right)$$

where $\Delta p$ is the pressure difference between the two static fluids, i.e. water and air, $R_1$, $R_2$ are the principal radii of curvature, $\gamma \cong 0.072\ N/m$ is the surface tension of water. When the film is adhered to the

wet soil, the thickness $h_l$ of the liquid bridge connecting the soil and the film is generally small (e.g. at the magnitude below 5 mm), in which case $\frac{1}{R_1} \to 0$ and $\frac{1}{R_2} \to \frac{2}{h_l}$. Then the applied pressure on the strip can be calculated as

$$p_{capillary} = \frac{2\gamma}{h_l} \geq 28.8 \text{ Pa}, \qquad \text{when } h_l \leq 5 \text{ mm}$$

This calculated value has the same magnitude as the capillary pressure obtained from the peeling experiment.

## Wing deformability analysis

The main goal of the analysis is to illustrate, to which degree the wing film would deform, when endured forces at different magnitudes. The filmy wing can be seen as being composed of a series of dense, elongated strips connected on their sides (fig. S7A). Each strip consists of a vein-like structure situated at the center and two symmetrical thin films on each side. Since the width of the strip along its length direction varies insignificantly, we regard each strip has uniform width in the subsequent analysis (fig. S7B). To analyze the bending deformability of the wing film, we consider a general case, when the wing is loaded with uniformly distributed pressure, which value should be the same as the pressure the seed may endured in natural scenarios, and which direction is perpendicular to the film surface.

Before the theoretical model is built, we adopt the finite element analysis to illustrate how the specialized structure and heterogeneous material composition would affect wing performance. Based on SEM observations, geometry models of a 500×500 μm wing section consisting of 20 connected 25×500 μm strip units are built using COMSOL Multiphysics (Version 6.0) (fig. S7B). Each unit comprises a cylindrical rod covered by a 0.2 μm-thick film layer, simulating the vein structure. At each side of the vein, a thin film with a thickness of 0.4 μm and a width of 25 μm extends horizontally outwards. All materials used in the wing structure are assumed to be isotropic. Young's moduli tested by AFM are assigned to the cylindrical rod (280 MPa) and the other film part (3925 MPa). For most materials, the Poisson's ratio falls within a narrow band from approximately 0.25 to 0.33 and hence an estimate of 0.30 was adopted for the Poisson's ratio of the model[8]. All displacements and rotations at one side of the model were constrained to zero, while the other three sides were set free. The deformation of the wing under bending was assessed by applying uniform pressure on the upper surface of the wing section. (fig. S7C, D). A mesh convergence test

was conducted, and the mesh set with a relative difference of less than 0.1% compared to a more densely refined mesh set was selected for use.

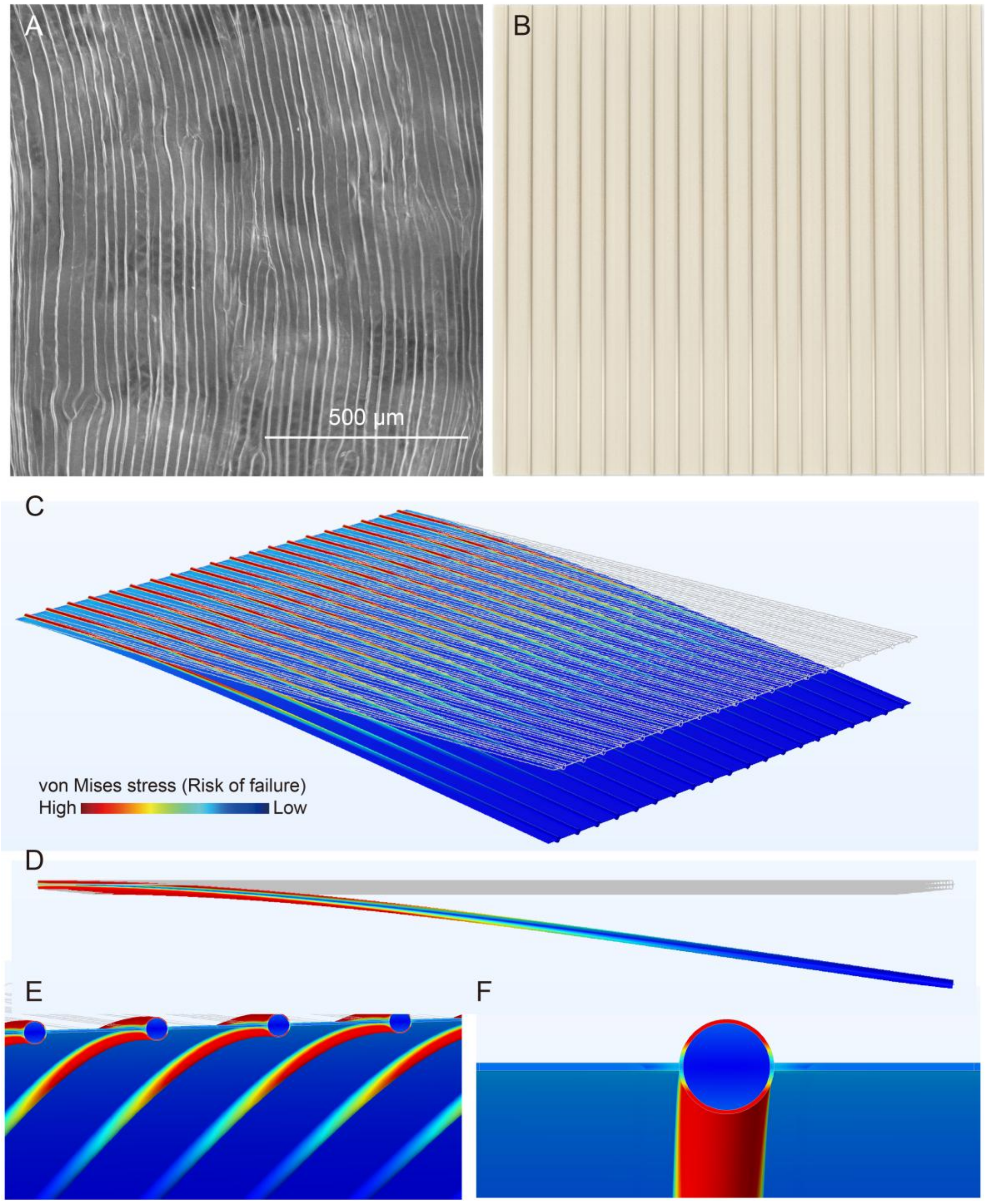


**Figure S7**. (A) SEM image showing a magnified view of the seed wing surface. (B) Geometric model consisting of 20 connected strips, simulating the structure of the filmy wing region. All displacements and rotations at one side of the model are constrained to zero, while the other three sides remain free. (C) Contour plot displaying the deformed shape from the finite element analysis under a uniformly distributed pressure on the surface. The color represents the value of von Mises stress endured by the structure. The grey mesh represents the initial undeformed shape of the film. (D) Lateral profile of the deformed shape

of the film. (E) Magnified view at the fixed end of the film. (F) Further magnified view at the fixed end of the film, illustrating the stress distribution in the cross-section of one strip unit.

The stress distribution observed in the cross-section of the FEM results indicates that the vein structure primarily bears the pressure during bending, while the contribution of the flattened film in terms of resisting deformation is negligible (fig. S7E, F). At the connected boundaries of adjacent strips, only tangential tensile forces (perpendicular to the loading direction) are present in the film, which do not influence deflection within the bending plane. Therefore, we propose that the deformed profile of a single strip structure under uniform load can be used to describe the deflection of the filmy wing structure under the same uniform pressure. We will compare the model's predictions with the finite element results of the wing structure to demonstrate the strip model's accuracy in capturing the bending deformation of the filmy wings.

**Bending stiffness**

In the following theoretical model of a single strip unit, we simplify the strip vein as a cylindrical rod with diameter $d$ wrapped by a thin film with a thickness of $h_f/2$, resulting in the total vein diameter of $D = d + h_f$, where $h_f$ is the thickness of the side films (fig. S8). The width of the entire strip is $b$, and the net width of two side films is $b_f = b - D$. The total length of the strip unit is $l$.

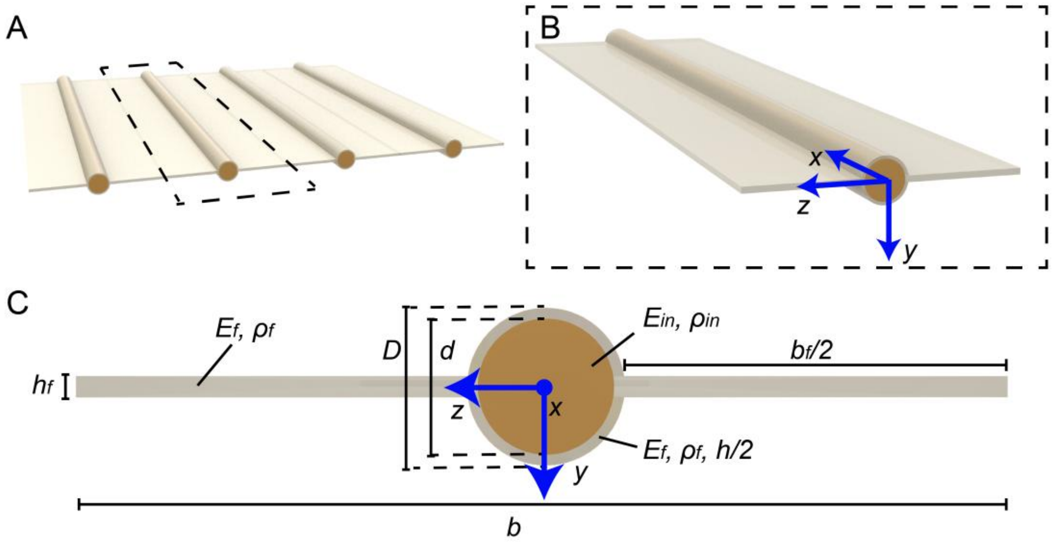


**Figure S8**. (A) Cross-sectional view of the geometrically simplified wing containing four units of the wing strips used for theoretical modeling. (B) Single strip unit showing the reference frames for modeling. (C) The main physical parameters in the cross-section of the strip structure.

Let us consider a single strip with one end ($x$=0) fixed and the other end ($x$=1) entirely free, subjected to a uniformly distributed pressure $p$. As illustrated in fig. S8, a reference coordinate system is set up for a single strip unit. For any point within the cross-section of the strip unit, the geometric relationship is given by:

$$\varepsilon = y/\rho \tag{S1}$$

where $y$ denotes the distance from a point within the cross-section to the neutral plane, $\varepsilon$ is the strain at this point in the bending direction, and $\rho$ is the radius of curvature of the neutral plane. Given that the side films are very thin, we assume that their lateral deformations during bending have minimal impact on adjacent strips. In this framework, we apply a one-dimensional Hooke's law to the strip cross-section.

$$\sigma = E\varepsilon \tag{S2}$$

where $E$ is the Young's modulus of a point in the cross-section. Substituting equation (S1) into equation (S2) gives

$$\sigma = E\frac{y}{\rho} \tag{S3}$$

The elastic moduli of the vein inner rod and strip film are $E_{in} = 280$ MPa and $E_f = 3925$ MPa, respectively, which values were determined by atomic force microscopy test.

Within the bending cross-section plane, the internal forces form a bending moment $M_z$, which is equivalent to the externally applied torque $M$:

$$M_z = M = \int_A y\sigma \,\mathrm{d}A \tag{S4}$$

Substituting equation (S3) into equation (S4) and adopted the experimental-derived modulus at different region, gives:

$$\begin{aligned} M &= \int_A y\sigma \,\mathrm{d}A = \int_{A1} y\sigma \,\mathrm{d}A + \int_{A2} y\sigma \,\mathrm{d}A + \int_{A3} y\sigma \,\mathrm{d}A \\ &= \frac{E_f}{\rho}\int_{A1} y \,\mathrm{d}A + \frac{E_f}{\rho}\int_{A2} y \,\mathrm{d}A + \frac{E_{in}}{\rho}\int_{A3} y \,\mathrm{d}A \\ &= \frac{E_f}{\rho}\frac{(b-h-d)h^3}{12} + \frac{E_f}{\rho}\frac{\pi}{64}(D^4 - d^4) + \frac{E_{in}}{\rho}\frac{\pi}{64}d^4 \\ &= \frac{1}{\rho}\left\{\left[\frac{(b-h-d)h^3}{12} + \frac{\pi}{64}(D^4 - d^4)\right]E_f + \frac{\pi}{64}d^4 E_{in}\right\} = \frac{B}{\rho} \end{aligned} \tag{S5}$$

where $A_1$, $A_2$, and $A_3$ represent the cross-sectional areas of the side films, vein covering film, and

vein inner rod, respectively. $B$ is the equivalent bending stiffness of the strip unit, which writes:

$$B(b,d,h) = \left[\frac{(b-h-d)h^3}{12} + \frac{\pi}{64}(D^4 - d^4)\right]E_f + \frac{\pi}{64}d^4E_{in}$$
$$= \left[\frac{(b-h-d)h^3}{12} + \frac{\pi}{64}(h^4 + 6d^2h^2 + 4dh^3 + 4d^3h)\right]E_f + \frac{\pi}{64}d^4E_{in} \tag{S6}$$

clearly, $b, d, h$ are the control geometrical parameters of the bending stiffness.

For small deflection $w$, equation (S5) can be rewritten as

$$\frac{M}{B} = \frac{1}{\rho} \cong \frac{\mathrm{d}^2w}{\mathrm{d}x^2} \tag{S7}$$

Integrating equation (S7) with respect to $x$ twice and applied the boundary condition of $w|_{x=0} = 0$, $\left.\frac{\mathrm{d}w}{\mathrm{d}x}\right|_{x=0} = 0$, yields the deflection curve equation:

$$w = \frac{qbx^2}{24\,B}(x^2 - 4lx + 6l^2) \tag{S8}$$

where $q$ $(\mathrm{N/m^2})$ is the uniformly distributed pressure on the strip. The deflection of the strip predicted by equation (S8) at a given pressure perfectly matches the deflection of the 20 strips-connected wing model calculated by FEM (fig. S9), suggesting the bending stiffness we derived in equation (S6) is accurate and the theoretical model should be appropriate for estimating the deformability of the wing.

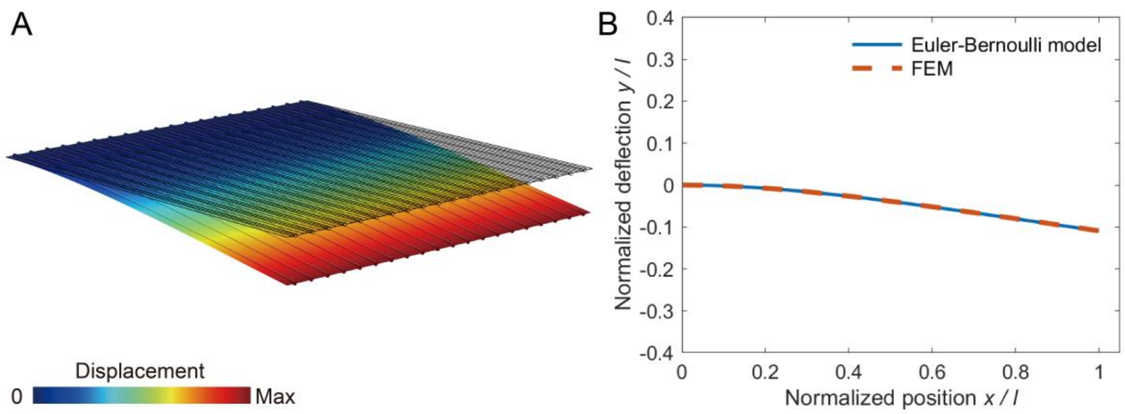


**Figure S9**. (A) FEM result showing small deflection of the wing section. (B) Comparison of the small wing deflection profile predicted by the FEM result and the Euler-Bernoulli model, respectively.

### Absolute Nodal Coordinate Formulation model

In the ANCF model built in Methods and Materials, the form of the nodal coordinate vector $\mathbf{e}$ and the element shape function matrix $\mathbf{S}(x)$ are listed as follows:

The global position vector of an arbitrary point $P$ on the neutral axis of the beam element is written as

$$\mathbf{r} = [r_1, r_2, r_3]^T = \mathbf{S}(x)\mathbf{e}$$

Here, $\mathbf{e}$ is the nodal coordinate vector:

$$\mathbf{e} = [e_1, e_2, e_3, e_4, e_5, e_6, e_7, e_8, e_9, e_{10}, e_{11}, e_{12}]^T$$

where

$$e_1 = r_{i1}, e_2 = r_{i2}, e_3 = r_{i3}, e_6 = r_{k1}, e_7 = r_{k2}, e_8 = r_{k3}$$

$$e_4 = \frac{\partial r_{i1}}{\partial x}, e_5 = \frac{\partial r_{i2}}{\partial x}, e_6 = \frac{\partial r_{i3}}{\partial x}, e_{10} = \frac{\partial r_{k1}}{\partial x}, e_{11} = \frac{\partial r_{k2}}{\partial x}, e_{12} = \frac{\partial r_{k3}}{\partial x}$$

which includes the global position coordinates $r_i(r_{i1}, r_{i2}, r_{i3})$, $r_k(r_{k1}, r_{k2}, r_{k3})$ and global slopes $\partial r_i/\partial x$, $\partial r_k/\partial x$ of the two nodes on a beam element, respectively. Here, $x$ is the local coordinate of the element in the undeformed configuration. In the absolute nodal coordinate formulation, the use of slopes instead of rotations allows the representation of complex shapes using a small number of elements. $\mathbf{S}(x)$ is the element shape function matrix, written as:

$$\mathbf{S} = \begin{bmatrix} s_1 & 0 & 0 & s_2 l & 0 & 0 & s_3 & 0 & 0 & s_4 l & 0 & 0 \\ 0 & s_1 & 0 & 0 & s_2 l & 0 & 0 & s_3 & 0 & 0 & s_4 l & 0 \\ 0 & 0 & s_1 & 0 & 0 & s_2 l & 0 & 0 & s_3 & 0 & 0 & s_4 l \end{bmatrix}$$

where $s_1 = 1 - 3\xi^2 + 2\xi^3, s_2 = \xi - 2\xi^2 + \xi^3, s_3 = 3\xi^2 - 2\xi^3, s_4 = \xi^3 - \xi^2, \xi = x/l$.

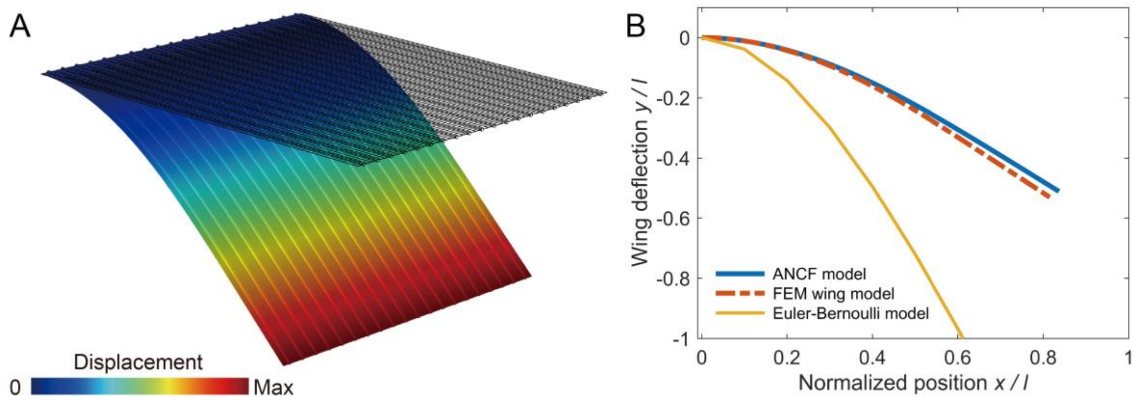


**Figure S10**. (A) FEM result showing large deflection of the wing section. (B) Comparison of the large wing deflection profiles predicted by the ANCF model, FEM result, and the Euler-Bernoulli model, respectively, under the same evenly distributed pressure.

## Wing toughness analysis

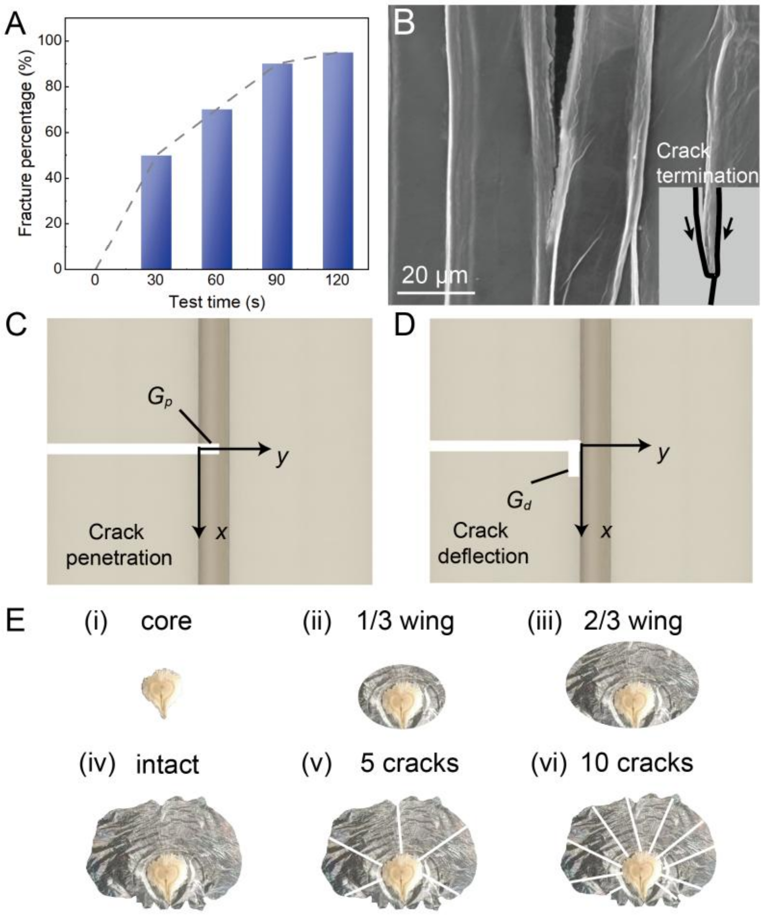


**Figure S11**. Seed fracture patterns and analytical model. (A) Percentage of the fractured wing seed against test time in the turbulent airflow experiments. (B) Damaged wing with a crack terminated by the intersection of two veins. (C) Schematic showing a crack started from the film region penetrating the fiber region at a right angle. (D) Schematic showing a crack being deflected by the fiber region and propagating along the film-fiber interface. (E) Different fracture patterns used in dispersal performance test, including (i) the seed only left with the core, (ii) the seed with 1/3 wing area remaining, (iii) the seed with 2/3 wing area remaining, (iv) intact seed, (v) the seed with 5 radial cracks on the wing, (vi) the seed with 10 radial cracks on the wing.

Assume that a crack propagates on a thin plate under a symmetric load from upper and lower boundaries, approaching the film-fiber interface at a right angle. The crack can either propagate ahead into the fiber (fig. S11C) or be deflected along the interface and continue to propagate on the film (fig. S11D).

The materials on either side of the interface are taken to be elastic and isotropic with Young's modulus $E_{in}$ and $E_f$ and Poisson's ratio $\nu_{in}$ and $\nu_f$, where the subscripts $f$ and $in$ refer to the film and the inner fiber, respectively. In plane stress problem, the elastic mismatch between two materials is described by Dundurs' parameters [9].

$$\alpha = \frac{E_{in} - E_f}{E_{in} + E_f}$$

$$\beta = \frac{(1 - \nu_{in})/E_{in} - (1 - \nu_f)/E_f}{2(E_{in} + E_f)}$$

**(1) The crack tip penetrating the interface**

The semi-infinite reference crack with length $a \to 0$ is perpendicular to the fiber with its tip at the interface. A symmetric loading with respect to the crack plane is applied and the stress distribution ahead of the crack in fiber (fig. S11C) is characterized by [10,11]

$$\sigma_{xx}(0, y) = k_1(2\pi y)^{\gamma}$$

where $k_1$ is the amplitude factor and $\gamma$ is the stress singularity assuming real values in the range: $(-1 < \gamma < 0)$.

In the case of penetration, the stress state at the advancing tip is pure mode I (tensile stress pulls the crack surfaces apart). By dimensional considerations, its stress intensity factor must depend on $k_1$ and $a$ according to

$$K_{\mathrm{I}} = c k_1 a^{1/2+\gamma}$$

where $c$ is a dimensionless function of $\alpha$ and $\beta$. In plane stress problem, the energy release rate is given by

$$G_p = \frac{K_{\mathrm{I}}^2}{E_f} = \frac{c^2 k_1^2 a^{1+2\gamma}}{E_f}$$

**(2) Crack tip being deflected along the interface**

The stress distribution on the interface directly ahead of the right-hand tip of the deflected crack (fig. S11D) is characterized by [12]

$$\sigma_{yy}(x,0) + \mathrm{i}\sigma_{xy}(x,0) = (2\pi r)^{-1/2} K r^{\mathrm{i}\varepsilon}$$

Where $K$ is the complex stress intensity factor for the interface crack, $r = x - a$, $i = \sqrt{-1}$, and

$$\varepsilon = \frac{1}{2\pi} \ln\left(\frac{1-\beta}{1+\beta}\right)$$

In this case, dimensional considerations require

$$K = k_1 a^{\frac{1}{2}+\gamma}\left[d a^{\mathrm{i}\varepsilon} + e a^{-\mathrm{i}\varepsilon}\right]$$

where $d$ and $e$ are dimensionless complex constants. The energy release rate of the deflected crack is [12]

$$G_d = \frac{H}{4\cosh^2 \pi\varepsilon} |K|^2 = \frac{H}{4\cosh^2 \pi\varepsilon} k_1^2 a^{1+2\gamma}[|d|^2 + |e|^2 + 2R_e(de)]$$

where $H = 2\left(1/E_f + 1/E_{in}\right)$.

### (3) Crack deflection criteria

The critical ratio of the energy release rate of the deflected crack and the penetrating crack $G_d/G_p$ is given by

$$G_d/G_p = \frac{HE_f}{4\cosh^2 \pi\varepsilon} \frac{|d|^2 + |e|^2 + 2\mathrm{Re}(de)}{c^2}$$

which is independent of $a$ and $k_1$. Thus, the relative tendency of a crack to be deflected by the interface or to pass through it can be assessed using this ratio. The function $c$ for the case of the penetrating crack and for $d$ and $e$ for the case involving deflected crack can be solved through integral equation methods [13].

For most materials, Poisson's ratio falls within a narrow band from approximately 0.25 to 0.35 [14]. Regarding this range, an estimate of 0.30 can be made for the Poisson's ratio for both the film and the inner fiber, i.e., $\nu_{in} = \nu_f = 0.3$. Then in our case, $\alpha = -0.87$ and $\beta = 2.76 \cdot 10^{-7}$. As $\alpha$ dominates $G_d/G_p$ and the small $\beta$ value usually has a negligible effect [13], the ratio $G_d/G_p$ can be plotted as a function of $\alpha$ with $\beta = 0$ (fig. 4I).

According to the criteria, the approaching crack is likely to be deflected into the interface if

$$\frac{\Gamma_f}{\Gamma_{in}} < \frac{G_d}{G_p}$$

where $\Gamma_f$ and $\Gamma_{in}$ are the toughness of the film and the inner fiber, respectively.

## Physical parameter analysis

We next explored how the geometrical parameters of the wing strip have been shaped to their current configuration. From the previous analysis, we can infer that the fiber diameter $d$ should be specialized to enhance toughness, while the strip length $l$ determining the wing span should be set for providing sufficient aerodynamic force. Hence, our focus shifts to investigating the strip width $b$ and the film thickness $h$, which could be optimized for enhancing the wing stiffness, reducing the weight, or promoting the materials efficiency when building the wing (fig. S12).

According to the equation (S8), the normalized maximum deflection at the free end is:

$$w_{max}/l = w(x = l) = \frac{ql^3}{8\,B_c}$$

where $B_c = B/b$ is the effective bending stiffness averaged by the strip unit width. The above equation indicates that the maximum deflection of the wing is inversely proportional to the effective bending stiffness, i.e. $w_{max}/l \sim 1/B_c$. Hence, a larger $B_c$ would enable the wing to deform less at a given load, facilitating the seed to expand its wing to sustain aerodynamic force.

The mass density $\rho_c$ $(\mathrm{kg/m^2})$ averaged in the strip cross-section can be written as:

$$\rho_c = \frac{\pi}{4}\frac{d^2}{b}\rho_{in} + \left[\frac{\pi}{4b}(h^2 + 2dh) + h(b - h - d)\right]\rho_f$$

where $\rho_{in}$ and $\rho_f$ are the density of the vein inner fiber and the film, respectively. The inner fiber composed of cellulose may have a density of $1450 - 1590\ \mathrm{kg/m^3}$ [1], and we use the median value $\rho_{in} = 1500\ \mathrm{kg/m^3}$ as estimation. The film that composed of lignin may have a density of $1200 - 1250\ \mathrm{kg/m^3}$ [1], and we use the median value $\rho_f = 1225\ \mathrm{kg/m^3}$ as estimation.

The material efficiency refers to the mass of the material used to achieve the required performance of a functional structure[15]. In our case, this can be expressed as the ratio of the effective bending stiffness to the mass density $B_c/\rho_c$.

The theoretical model shows that increasing the film thickness $h$ leads to a rapid increase in both effective bending stiffness $B_c$ and averaged mass density $\rho_c$ of the wing (fig. S12B, C), and the synergistic effect leads to significant promotion of the material efficiency $B_c/\rho_c$ of the wing (fig. S12D). The same effects of increasing the wing stiffness and the mass density can be achieved by decreasing the strip width $b$, which means densifying the strip array at a certain wing area (fig. S12B, C). If comparing thickening the

film and densifying the strip array together, increasing the film thickness $h$ could be a more efficient way to enhance the bending stiffness, but would also result in a higher gain in weight as a side effect (fig. S12D). Considering that the strip film in the real seed is only composed of a single-cell-layer film, being the thinnest form that a single plant cell can achieve, we believe that nature prioritizes weight reduction rather than achieving a high stiffness in a most efficient way for the wing. Although the seed could also reduce the wing's weight by adopting a sparser strip array, this would be a less efficient approach to maintain stiffness (fig. S12). Moreover, a wider interconnected strip film would inevitably have more potential defects for crack formation. Therefore, the current strip width likely represents an optimal balance among stiffness, weight, and toughness.

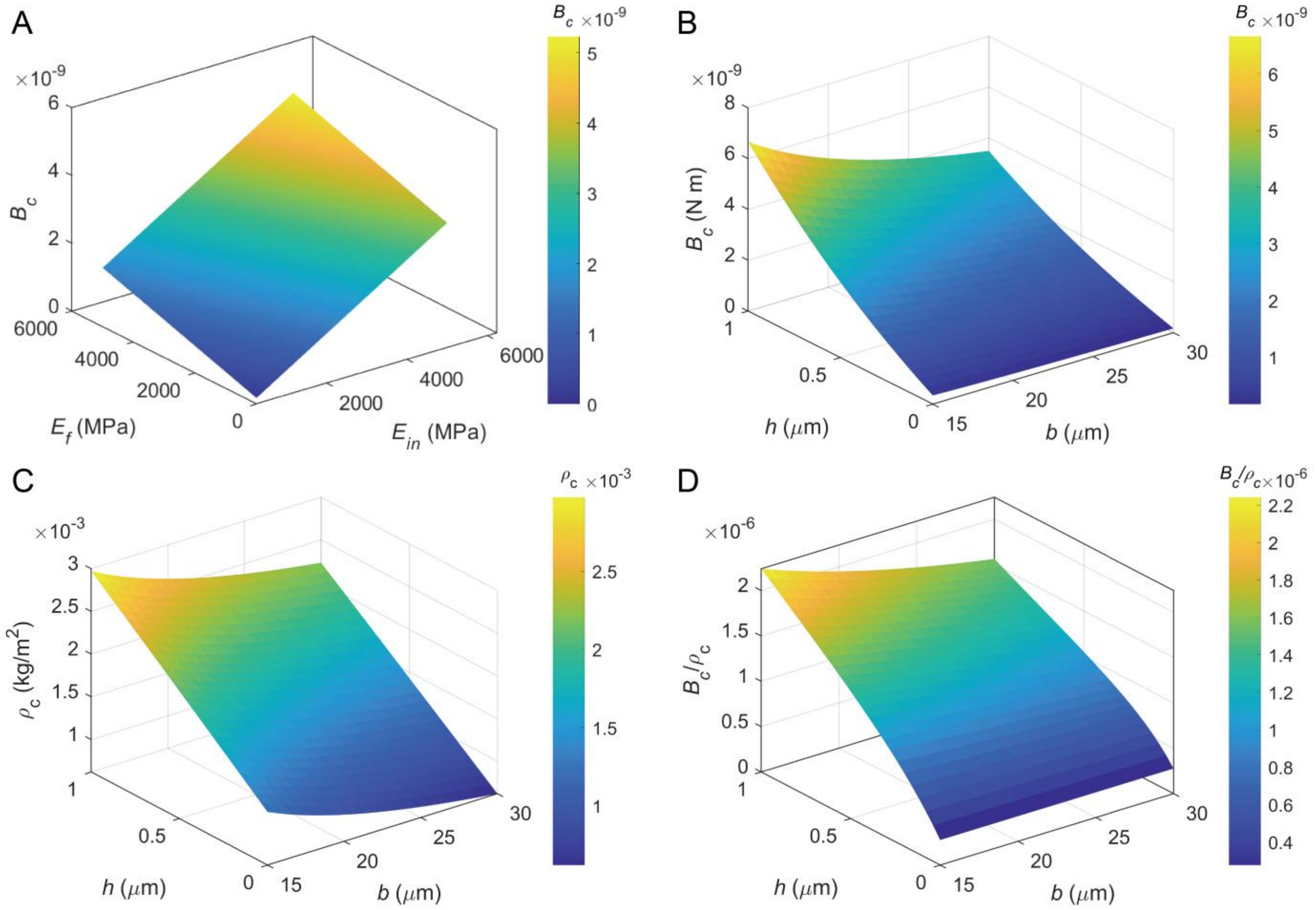


**Figure S12**. Mechanical parameter analysis. (A) Variation of the effective bending stiffness $B_c$ with respect to the Young's modulus of $E_f$ and $E_{in}$, respectively. (B) Variation of $B_c$ with respect to the film thickness $h$ and the strip width $b$, respectively. (C) Variation of the mass density $\rho_c$ with respect to $h$ and $b$, respectively. (D) Variation of the material efficiency $B_c/\rho_c$ with respect to $h$ and $b$, respectively.

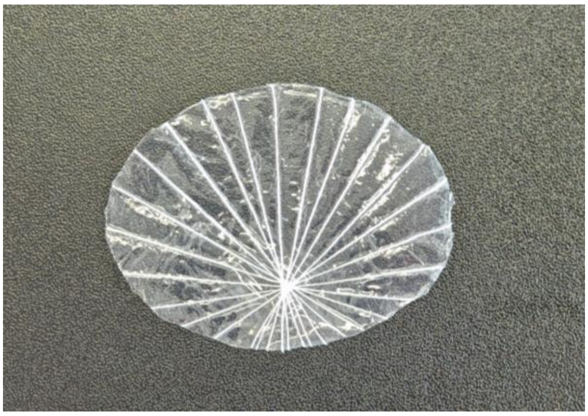

**Figure S13**. Biodegradable micro-fliers fabricated by the Polyvinyl Alcohol (PVA) water-solvable fibers (diameter: 180 μm) and Polybutylene Adipate Terephthalate (PBAT) cover film (thickness: 10 μm).

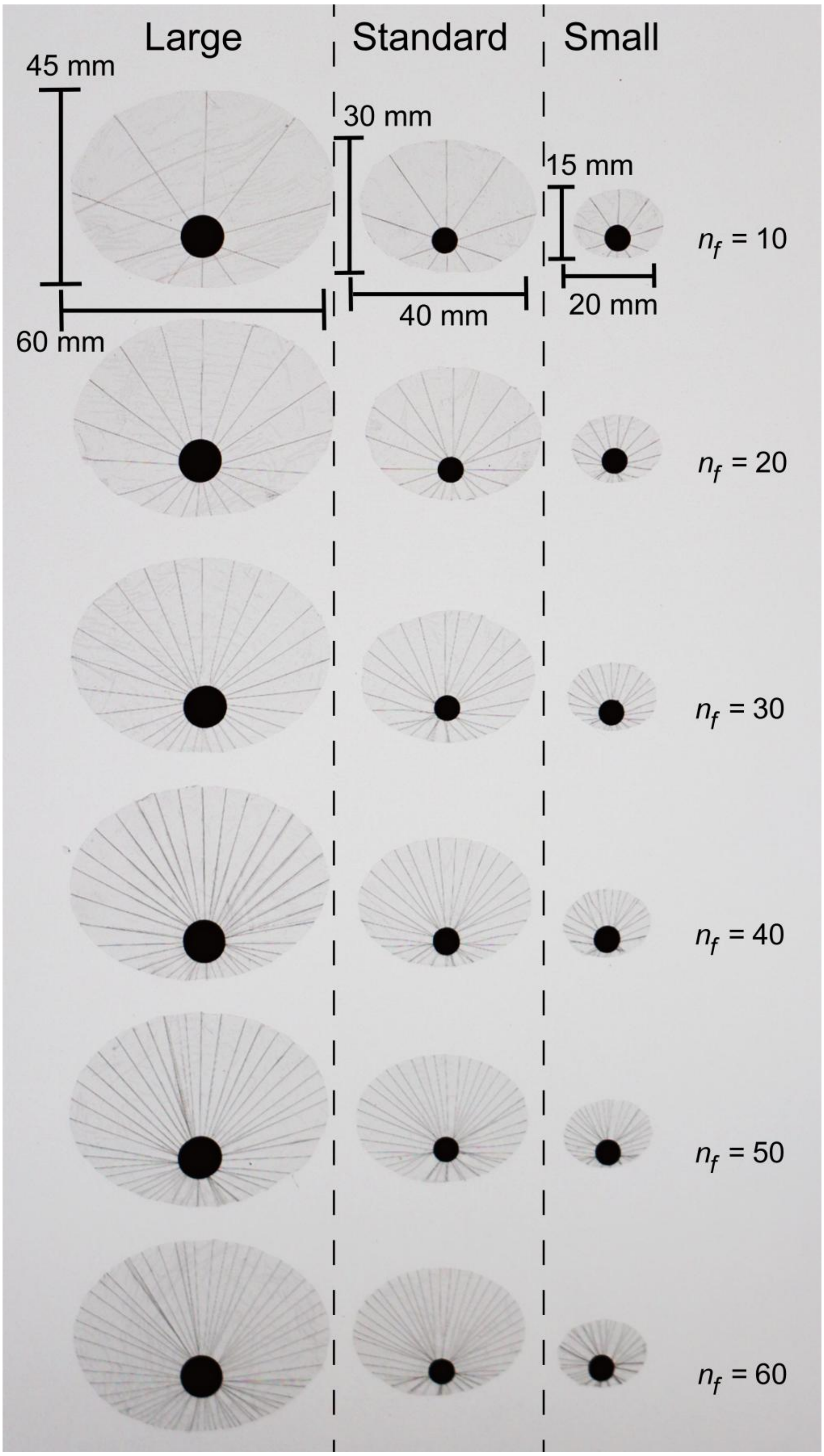


**Figure S14**. Image showing three sizes of the bionic micro-fliers with the number of the embedded fibers $n_f$ changed from 10 to 60.

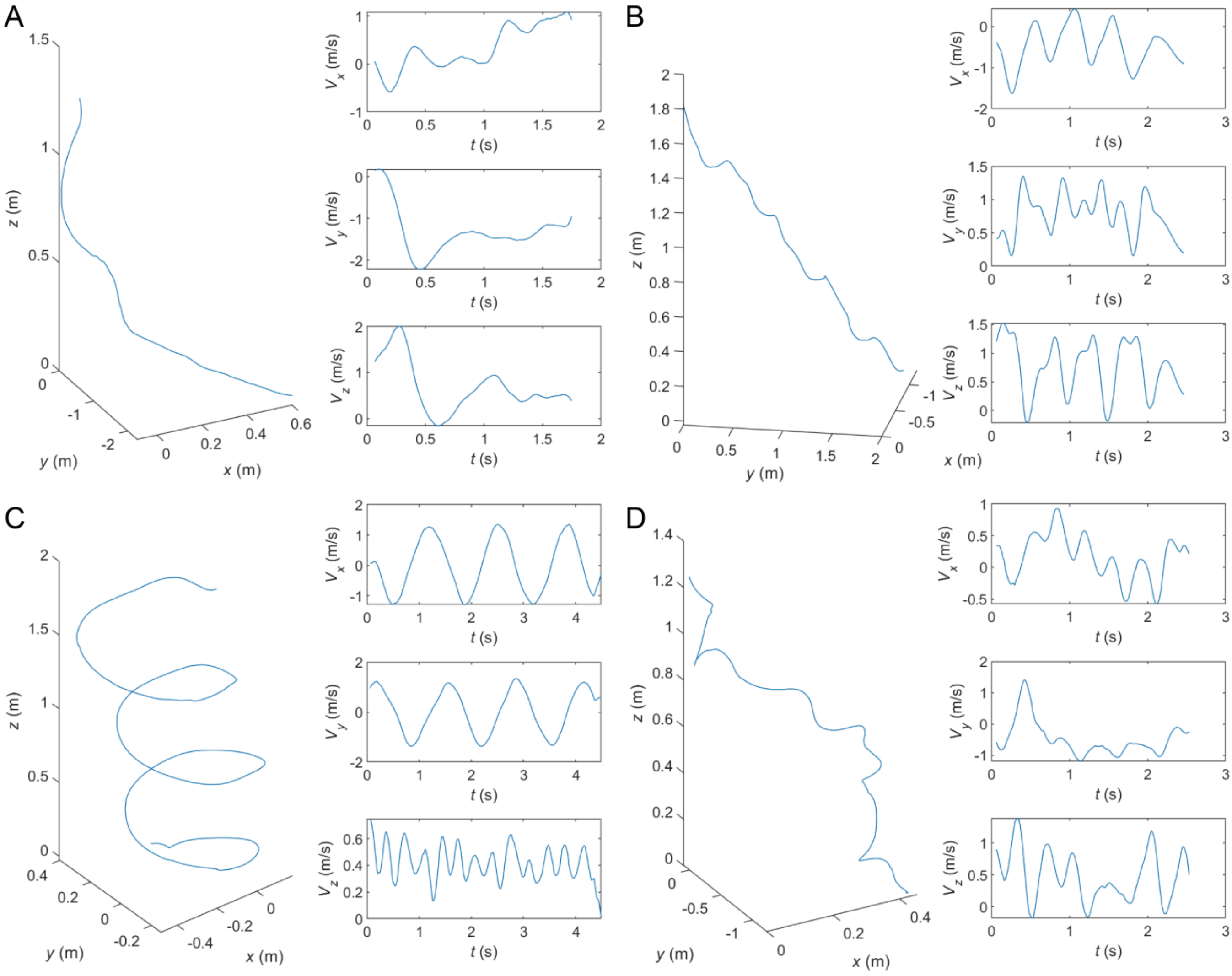


**Figure S15**. Spatial trajectories and velocities of typical flight patterns selected from fig. 5E. (A) Gliding pattern. (B) Fluttering pattern. (C) Rotating falling pattern. (D) Mixed pattern.

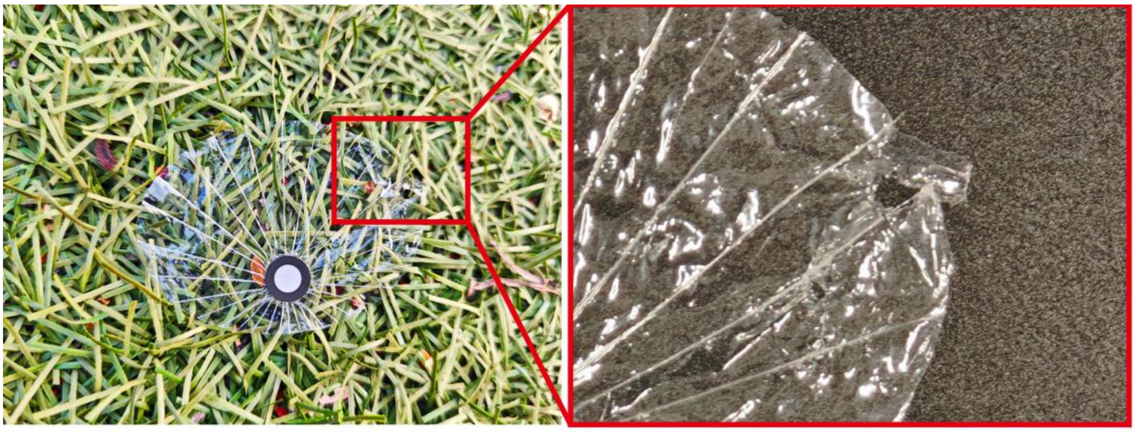

**Figure S16**. Sample damaged by drone propeller showing a fractured pattern at the micro-flier edge. The crack on the edge could be terminated by the embedded fiber, resembling to the function of the vein fiber of the natural seed.

## Legends for video S1 to S7

Video S1. Natural seed flight in the turbulent airflow

Video S2. Deformation of the seed wing, when rotating in the turbulent airflow

Video S3. Seed wing folds upon touching a droplet

Video S4. Seed wing fractured in the turbulent airflow

Video S5. Natural seed falling

Video S6. Bionic micro-flier falling

Video S7. Bionic micro-flier flight in the turbulent airflow

# References:


1. Gibson LJ. The hierarchical structure and mechanics of plant materials. *J R Soc Interface* 2012; **9**: 2749–66.

2. Ursache R, Andersen TG, Marhavý P *et al.* A protocol for combining fluorescent proteins with histological stains for diverse cell wall components. *Plant J* 2018; **93**: 399–412.

3. Dickinson MH, Götz KG. Unsteady aerodynamic performance of model wings at low Reynolds numbers. *J Exp Biol* 1993; **174**: 45–64.

4. Li H, Goodwill T, Wang ZJ *et al.* Centre of mass location, flight modes, stability and dynamic modelling of gliders. *J Fluid Mech* 2022; **937**.

5. Andersen A, Pesavento U, Wang ZJ. Unsteady aerodynamics of fluttering and tumbling plates. *J Fluid Mech* 2005; **541**: 65–90.

6. Kreitschitz A, Kovalev A, Gorb SN. Plant seed mucilage as a glue: Adhesive properties of hydrated and dried-in-contact seed mucilage of five plant species. *Int J Mol Sci* 2021; **22**: 1443.

7. Kreitschitz A, Gorb SN. How does the cell wall 'stick'in the mucilage? A detailed microstructural analysis of the seed coat mucilaginous cell wall. *Flora* 2017; **229**: 9–22.

8. Flannigan WC. Finite Element Modeling of Arthropod Exoskeleton. 1998.

9. Dundurs J. Discussion:"Edge-bonded dissimilar orthogonal elastic wedges under normal and shear loading." *J Appl Mech* 1969; **36**: 650–2.

10. Ming-Yuan H, Hutchinson JW. Crack deflection at an interface between dissimilar elastic materials. *Int J Solids Struct* 1989; **25**: 1053–67.

11. Evans AG, He MY, Hutchinson JW. Interface debonding and fiber cracking in brittle matrix composites. *J Am Ceram Soc* 1989; **72**: 2300–3.

12. Suo Z. Singularities, interfaces and cracks in dissimilar anisotropic media. *Proc R Soc Lond Math Phys Sci* 1990; **427**: 331–58.

13. Martínez D, Gupta V. Energy criterion for crack deflection at an interface between two orthotropic media. *J Mech Phys Solids* 1994; **42**: 1247–71.

14. Greaves GN, Greer AL, Lakes RS *et al.* Poisson's ratio and modern materials. *Nat Mater* 2011; **10**: 823–37.

15. Wegst UG, Ashby M. The mechanical efficiency of natural materials. *Philos Mag* 2004; **84**: 2167–86.
